# Hybrid photocatalytic-ozonation for wastewater remediation: mechanisms and synergistic effects

**DOI:** 10.1039/d6ra01948j

**Published:** 2026-05-06

**Authors:** M. S. Lawan, Mansour A. Balkhyour, Rajeev Kumar, M. A. Barakat

**Affiliations:** a Department of Environment, Faculty of Environmental Science, King Abdulaziz University Jeddah-21589 Saudi Arabia mababdullah1@kau.edu.sa mabarakat@gmail.com +966-0126400000; b Central Metallurgical R&D Institute Helwan 11421 Cairo Egypt

## Abstract

Hybrid photocatalytic ozonation (PC/O_3_) has emerged as one of the most powerful advanced oxidation processes (AOPs) for degrading highly recalcitrant organic contaminants in industrial wastewater. By integrating semiconductor photocatalysis with ozone-driven oxidation, PC/O_3_ systems unlock synergistic reaction pathways that significantly accelerate the production of hydroxyl radicals, suppress electron–hole recombination, and improve ozone utilization efficiency. This review presents a comprehensive and critical evaluation of recent advances in photocatalysis, ozonation, and their hybridization, with emphasis on mechanistic insights, catalyst engineering strategies, and operational factors governing PC/O_3_ hybrid performance. State-of-the-art developments in the strategies designed to enhance photocatalysts' visible-light harvesting, prolong carrier lifetimes, and strengthen ozone activation have been summarized. Key process parameters, including catalyst dosage, ozone concentration, pH, light intensity, pollutant chemistry, and reactor configuration, are systematically analysed to demonstrate their influence on radical generation kinetics, mineralization efficiency, and reaction energetics. Reported PC/O_3_ systems achieve 90–100% degradation and 60–90% TOC/COD mineralization for dyes, pharmaceuticals, and phenols, outperforming standalone AOPs in kinetics and stability. Reported PC/O_3_ systems consistently demonstrate superior degradation rates, higher TOC and COD removal, and improved stability compared to individual photocatalysis or ozonation. Despite this progress, challenges persist in catalyst deactivation, scale-up, ozone management, and economic feasibility. Emerging solutions, including advanced reactor engineering, solar-driven operation, robust catalyst architectures, and intensified mass-transfer designs, are highlighted as promising pathways toward practical industrial implementation. Collectively, this review provides an up-to-date, mechanistic, and application-oriented framework for the rational design of next-generation PC/O_3_ systems, offering a strategic pathway for translating laboratory innovations into sustainable industrial wastewater treatment technologies.

## Abstract 2

Hybrid photocatalytic ozonation (PC/O_3_) has emerged as one of the most powerful advanced oxidation processes (AOPs) for degrading highly recalcitrant organic contaminants in industrial wastewater.

## Graphical abstract

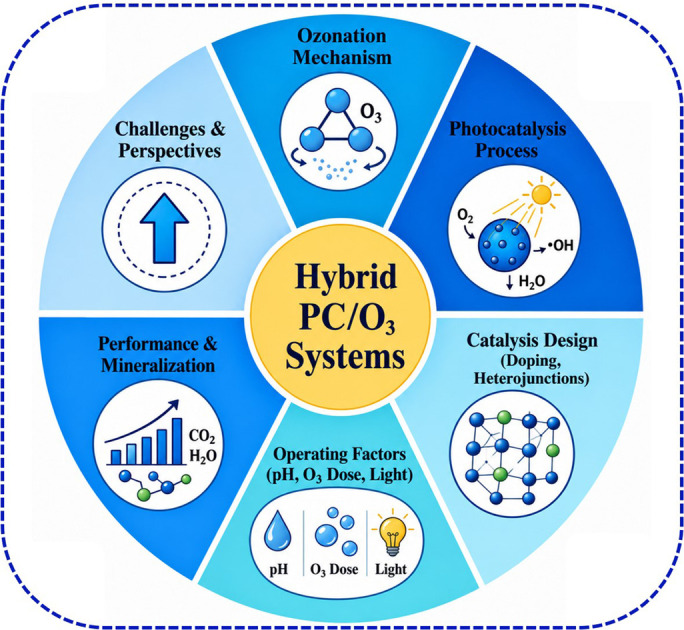

## 1. Introduction

**Fig. 1:**
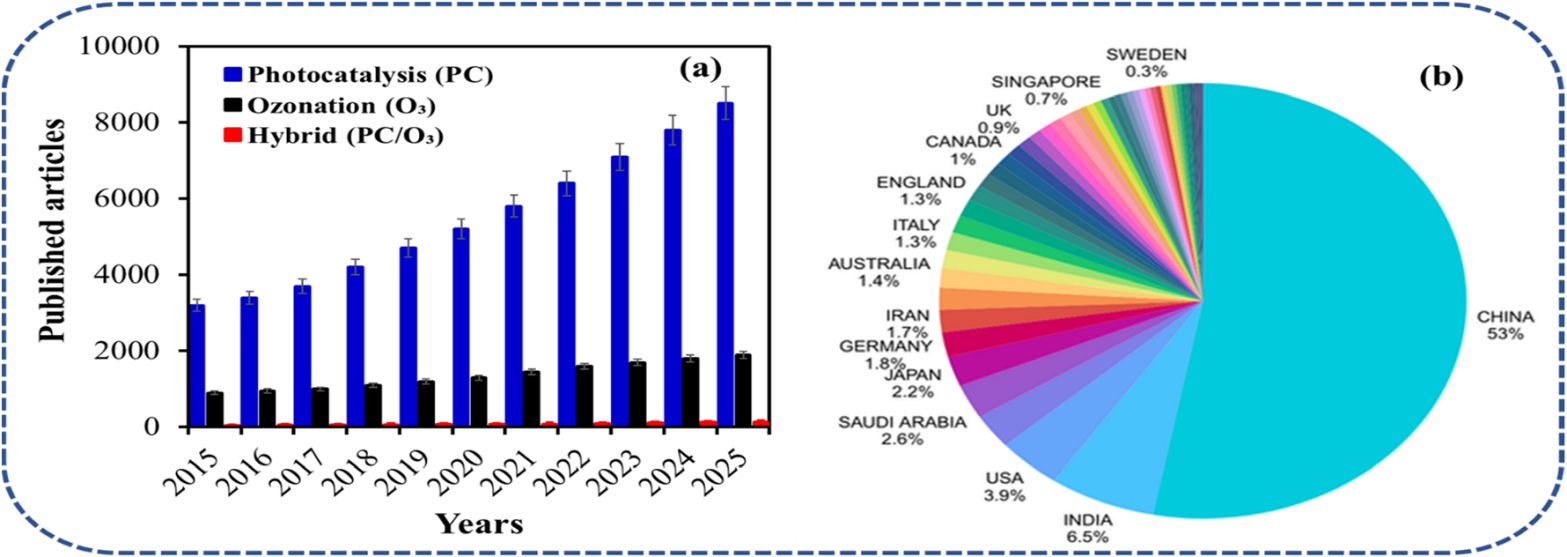
(a) Scopus publication trends in photocatalysis (blue), ozonation (black), and hybrid AOPs (red). (b) Top 50 countries by AOP output (Web of Science, 1950–Jan 30, 2026).

Water is an essential natural resource that supports the existence of all living organisms, driving the continuous growth of industrial activities for economic development. However, rapid population growth, leading to urbanization and industrialization, has led to unprecedented pressure on the world's available water resources, resulting in widespread water pollution issues. The disposal of untreated or inadequately treated industrial wastewater has introduced numerous organic and inorganic contaminants into aquatic ecosystems, posing serious threats to public health and general environmental integrity.^1^ Thus, industrial wastewater effluents such as pharmaceuticals, textiles, petrochemicals, and even food and beverages represent a particular concern due to the release of complex mixtures of recalcitrant organic compounds into water bodies.^2^ These wastewater contaminants are characterized by their potential toxicity, persistence, and resistance to conventional wastewater treatment methods.^3^ Most of these industrial wastewater typically exhibit high concentrations of organic matter, evidenced by elevated chemical oxygen demand (COD), often exceeding 20 000 mg O_2_ per L in some facilities.^4^ Conventional wastewater treatment technologies, such as adsorption, coagulation, flocculation, sedimentation, and bio-filtration, which primarily rely on physical separation, chemical precipitation, and biological degradation, have proven inefficient for the complete removal of these recalcitrant compounds. In this way, many studies have given attention to enhancing the efficiency of the conventional techniques or reducing costs and other limitations by integrating with more cheap, sustainable, and environmentally friendly methods. Fig. 1a highlights a Scopus data on the tremendous growth in AOP research, with photocatalysis publications surging ahead, while ozonation shows steady increase and hybrids accelerating post-2015.

This trend reflects AOPs' appeal for sustainable wastewater treatment, driven by solar potential and catalyst innovations, yet hybrid PC/O_3_ systems exhibit the steepest rise (>20× since 2010), signalling recognition of synergies for recalcitrant pollutants.^5^Fig. 1b effectively illustrates Web of Science analysis (1950–Jan 30, 2026) on the global research leadership in AOPs, revealing China as the dominant contributor with the highest publication volume, followed by the United States, India, and Iran among the top performers. The outcome of the analysis underscores East Asian and North American dominance, reflecting substantial investments in wastewater remediation technologies. Emerging nations like Saudi Arabia (including King Abdulaziz University) show rising output, signalling growing regional expertise in AOPs for industrial applications. The AOPs are regarded as a promising option to treat all forms of industrial wastewater organic contaminants, leading to their successful decomposition and mineralisation by an *in situ* generation of active oxidising reagents such as hydroxyl radical (˙OH), superoxide (˙O_2_^−^) ozonide (˙O_3_^−^), and photoproduced electron–hole pairs.^6,7^ The techniques regarded as generally comprising many individual process including ozone (O_3_), photocatalysis, hydrogen peroxide (H_2_O_2_), Fenton, ultraviolet (UV), and several hybrid processes like photocatalytic-ozonation (PC/O_3_), peroxone (O_3_/H_2_O_2_), O_3_/TiO_2_/H_2_O_2_ systems, UV light with peroxide (UV/H_2_O_2_), *etc.*

Among the different existing AOPs, the photocatalytic-ozonation process is emerging as a very promising hybrid alternative technique, featuring no sludge generation, and residual ozone also decomposes into water and oxygen.^8,9^ The particular advantage of this oxidation method applies to the complete mineralization of recalcitrant organic compounds or for improving the biological degradability.^10^ However, the hybrid PC/O_3_ system is still considered among the expensive treatment techniques for large scale application is not economically justifiable. In summary, the present review was written to highlight the benefits of the hybrid PC/O_3_ system as an advanced oxidation process for industrial wastewater treatment by exploring the most recently published studies in this field, the aim of which was to present the new concepts for a more effective approach to wastewater remediation. In this review, studies reported in the application of individual ozonation and photocatalysis processes, along with the influence of different experimental parameters on the decomposition of various wastewater organic contaminants, were highlighted. In-depth discussion on photocatalytic ozonation as a hybrid process, along with the reaction mechanisms, kinetics, synergistic effects, toxicity, and economic aspects, is well presented. Unlike existing reviews addressing individual AOPs or photocatalyst materials, this work delivers the first systematic quantitative comparison of ozonation, photocatalysis, and PCO_3_ for recalcitrant industrial wastewater treatment. We synthesize: (1) operating optima across processes (Table 5), (2) stability/economics benchmarking (Tables 6 and 7), and (3) scale-up limitations with practical design guidance (Section 5.3), filling critical gaps in comparative performance meta-analysis and industrial implementation feasibility.

### 1.1 Advanced oxidation processes (AOPs)

**Fig. 2:**
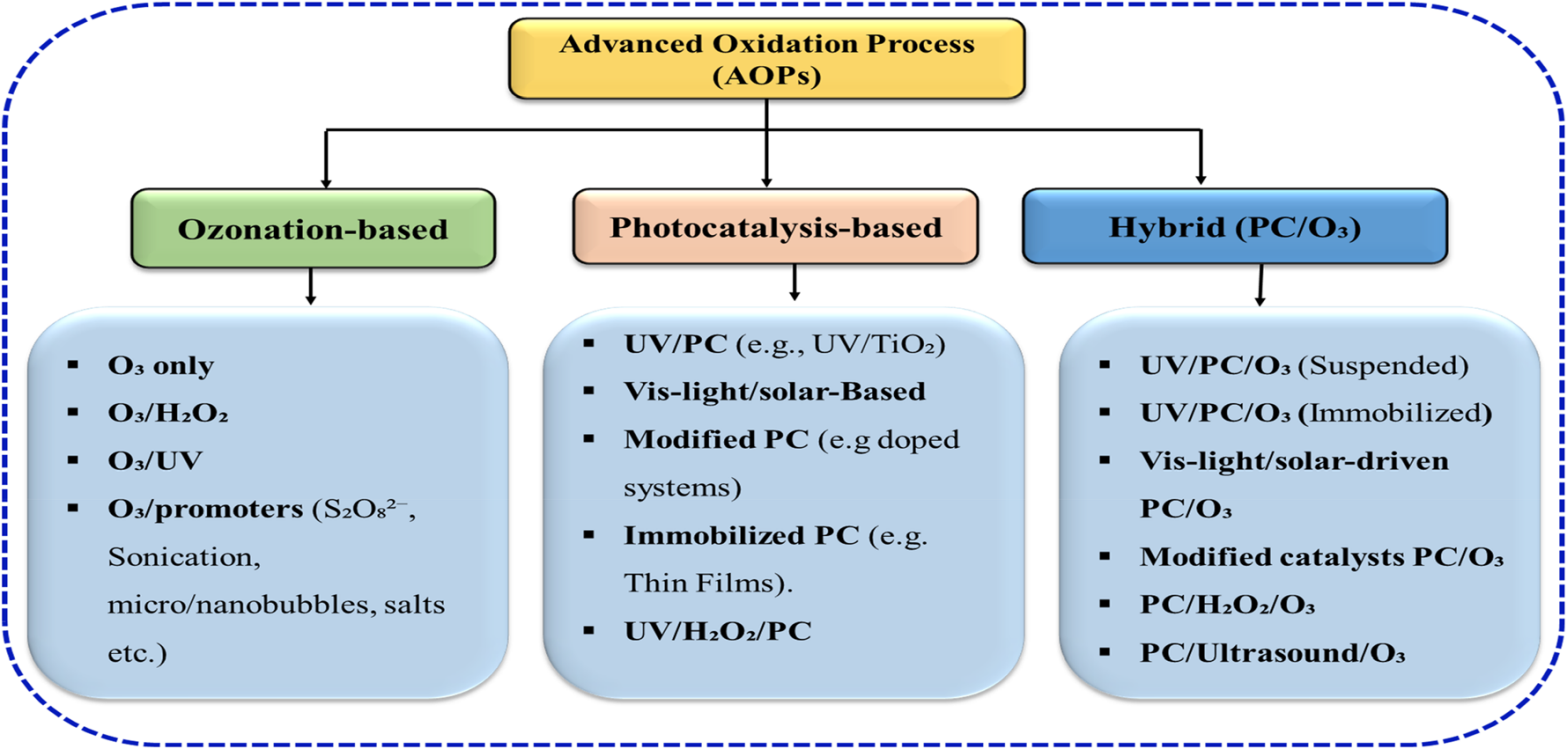
Overview of the advanced oxidation processes based on photocatalytic-ozonation.

The classification of AOPs is fundamental for understanding their applications, efficiency, and treatment limitations. Broadly, the AOPs are divided into either homogeneous or heterogeneous processes, in which the homogeneous category involves the oxidation reactions occurring in a single, typically aqueous, phase where soluble reagents interact under external radiation energy such as UV or visible light radiation.^11,12^ This category includes processes such as ozonation combined with H_2_O_2_, UV/H_2_O_2_, photo-Fenton oxidation, *etc.*^13^ Meanwhile, heterogeneous AOPs, on the other hand, rely on the application of a semiconducting photocatalysts such as TiO_2,_ which is activated by light energy to generate reactive radicals at its surface, promoting oxidation reactions.^14,15^ Additionally, the AOPs classification can also be based on the presence or absence of light, referring to a photochemical processes such as UV photolysis (utilizing a light source) and non-photochemical or literally “dark” oxidation processes such as ozonation, Fenton reactions without light, and ultrasonic cavitation.^16,17^Fig. 2 depicts a comprehensive overview of the AOPs classification organogram. This classification provides a practical framework for selecting treatment strategies based on reaction environment, energy input, and catalyst involvement. Homogeneous systems typically offer rapid kinetics due to uniform reactant distribution but may suffer from reagent consumption and sludge generation. In contrast, heterogeneous processes enable catalyst recovery and reuse, though they are often limited by mass transfer and light penetration constraints.^4,5^ The photochemical *versus* non-photochemical distinction further clarifies energy requirements and operational costs. Overall, these classifications emphasize the balance between efficiency, scalability, and sustainability, guiding the rational design and optimization of advanced oxidation systems for wastewater treatment.

## 2. Ozonation: fundamentals and recent advances

The AOPs by ozonation is a powerful oxidation process that harnesses the high reactivity of ozone (O_3_) to degrade a broad range of organic contaminants. The ozone molecule was first isolated and recognised as a distinct substance by Christian Friedrich Schönbein in 1840; however, its value as an effective oxidant and disinfectant for water treatment was not recognized until the late 19th and early 20th centuries.^18^ It is a soluble pale blue, triatomic molecule with a high oxidation potential (2.07 V).^19,20^ Unlike conventional oxidants such as chlorine, ozone does not leave harmful residues, decomposing to oxygen in a rapid reaction, thereby boosting the level of dissolved oxygen in the receiving water body. Thus, because of its high chemical instability, ozone must be generated onsite and is typically introduced into the wastewater through diffusers or injectors to allow rapid diffusion and contact with pollutants.^21,22^ These essential properties, along with high redox potential, significantly make the ozonation process an efficient oxidant to mineralize both organic and inorganic contaminants from wastewater effluents.^14^

### 2.1 Reaction mechanism in ozonation

As earlier mentioned, several recalcitrant organic compounds, including pharmaceuticals, chlorinated pesticides, solvents, and personal care products, can be oxidatively degraded through an ozonation mechanism.^23,24^ Inorganic contaminant matrices such as sulfide/bisulfide (H_2_S/HS), cyanide (CN) can also be oxidized. Furthermore, heavy metals oxidation is also possible with ozonation reaction, where metals like reduced iron [Fe(ii)], [As(iii)], and manganese [Mn(ii)]_*n*_ have been reported as successfully oxidized. In each case, the reaction mechanism usually results in the formation of either less harmful products [*e.g.*, oxygen (O_2_) nitrogen gas (N_2_), bicarbonate (HCO_3_) as in the case of cyanide and sulfide ozonation,^25^ or oxidized form of products such as As(vi) from the ozonation of As(iii) and/or precipitation Mn(iv) from Mn(ii), [Fe(iii) from Fe(ii)].^26^ The reaction of ozone with substances in aqueous medium (wastewater) has been established to occur in two distinct reaction kinetics as indicated in Fig. 4, *i.e.*, either through molecular ozone (direct reaction mechanism),^27^ or *via* the formation of secondary oxidants (hydroxyl radicals), which is referred to as indirect reaction pathways. In direct aromatic organic compounds ozonation such as phenol, it involves an initial electrophilic attack by the oxidant, followed by the loss of oxygen, resulting in hydroxylation of the aromatic ring (Fig. 5). The formation of the HO˙ group increases the reactivity toward electrophilic substitution reactions. Hence, it is possible that in a subsequent reaction, ozone may react with the aromatic ring *via* a 1,3-cycloaddition.^28^ In the indirect ozone oxidation pathway series of reactive oxygen species (ROS) like ˙HO, ˙O_2_^−^, H_2_O_2_, ˙HO_3_, are generated through a series of chain reactions by self-decomposition of ozone with aqueous contaminants.^29^ These hydroxyl radicals, which are capable of a non-selective oxidation of a broader range of organic contaminants, exhibit more reactive potential (about 1.35 times) than the molecular ozone. However, the reaction process is strongly pH-dependent and is preferentially accelerated under alkaline conditions and with integration of supporting agents/chemicals such as UV, sonication, and H_2_O_2_, *etc.*^30,31^ Thus, the ozonation process for organic wastewater degradation is usually designed in various configurations such as O_3_/UV, O_3_/H_2_O_2_, or even O_3_/H_2_O_2_/UV. The ozone-based oxidation is also often applied for the degradation of bulk organic load from industrial wastewater as an indicator for chemical oxygen demand (COD), total organic carbon (TOC), and dissolved organic carbon (DOC)] in industrial wastewaters. However, it is important to note that ozonation-based AOPs is not an effective process in the abatement of ammonia compounds (NH_3_/NH_4_^+^), selenite (SeO_4_^2−^) and other fully oxidized and per-halogenated compounds like ClO_4_.^32^

**Fig. 3:**
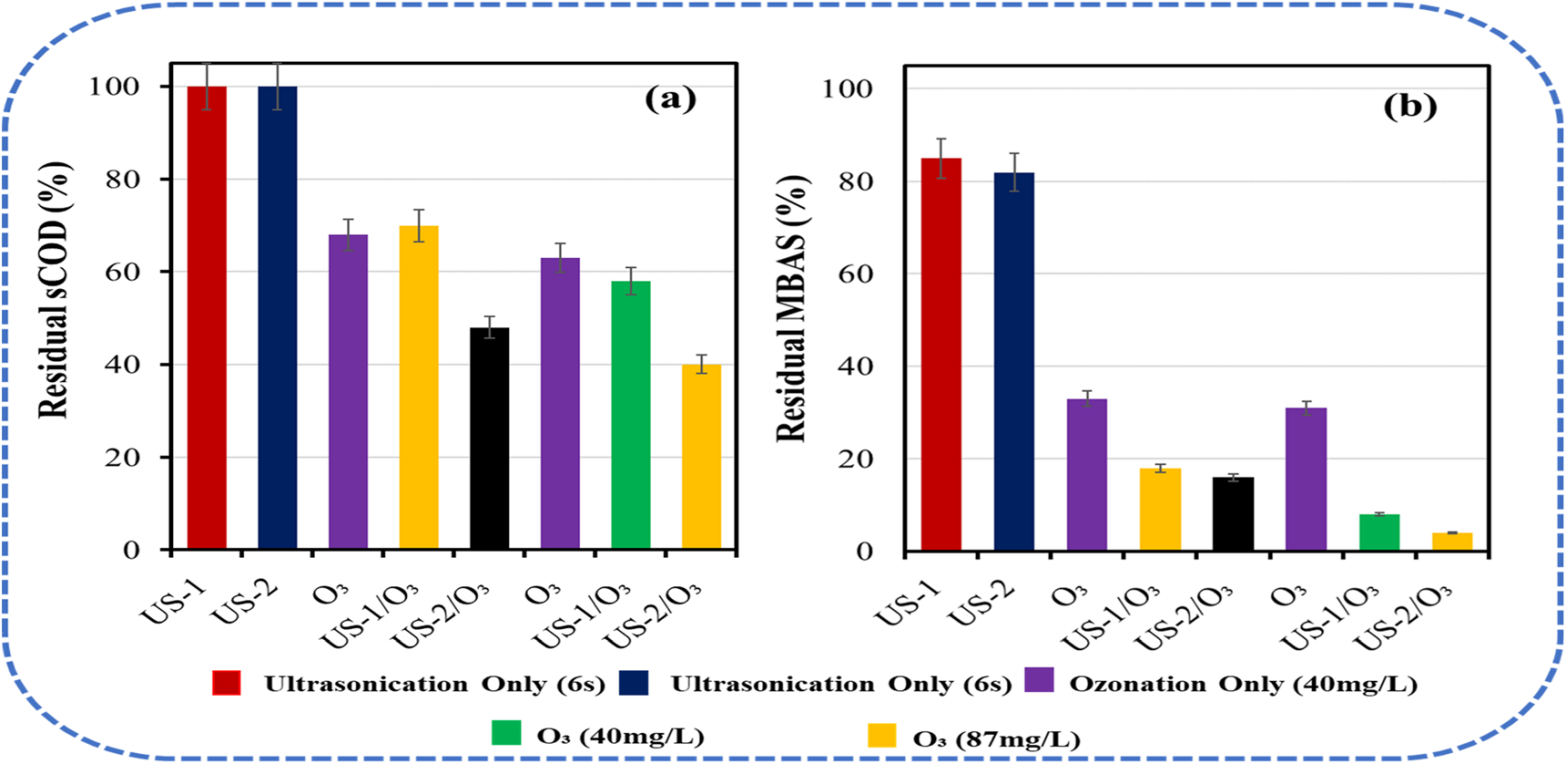
Effect of sonication on (a) COD and (b) MBAS removal by ozonation process, adapted with permission from [Springer Nature] ref. 33, copyright 2020.

Various studies, as indicated in Table 1, have reported the degradation performance of ozonation across a diverse range of organic contaminants and highlighted the effect of experimental conditions, such as pollutant type, ozone dose, contact time, pH, and hybridization with sonication/UV. For instance, Rossi *et al.*^33^ evaluated how ultrasonic pre-treatment combined with different ozone doses affects the removal of soluble chemical oxygen demand (sCOD) and anionic surfactants measured as methylene blue anionic surfactants (MBAS) from municipal primary effluent. In their work, ultrasonication alone at 6 s and 12 s (US-1 and US-2) showed negligible sCOD removal and limited MBAS abatement, with residual fractions close to the initial values (Fig. 3a and b). In contrast, ozonation alone at 40 mg L^−1^ and 87 mg L^−1^ reduced residual sCOD to about 70% and 68%, respectively, and residual MBAS to roughly 33% and 18%.

**Table 1:** Degradation of various organic wastewater using the advanced oxidation by ozonation process

Organic pollutants	Conc. (mg L^−1^)	Dose (mg L^−1^)	Time (min)	pH	Efficiency (%)	Ref.
Oxytetracycline	7.92 × 10^−3^	1.8 × 10^1^	15	∼7	89.32	34
Caffeine	9.6 × 10^−4^	1.8 × 10^1^	15	∼7	96.79	34
Ciprofloxacin	1.144 × 10^−2^	1.8 × 10^1^	15	∼7	97.75	34
Paracetamol	1.5117 × 10^−1^	1.8 × 10^1^	15	∼7	99.96	34
Methylene blue dye	1 × 10^1^	1 × 10^1^	20	∼7	>90	50
Acetamiprid and malathion	—	2 × 10^0^	10	7	84.80 and 70.08	51
Congo red	2.5 × 10^1^	2.5 × 10^1^	25	7	90%	40
Spirotetramat and acetamiprid	—	1 × 10^1^	10	7	100 and 46	52
Chlorpyrifos, cyhalothrin, and bifenthrin	—	1 × 10^1^	5	7	100 and 82.1	53
Congo red (azo dye)	5 × 10^1^	5 × 10^1^	60	9	98	42
Phenol	—	6 × 10^0^	20–60	7		44
Amoxicillin & paracetamol	1.827 × 10^2^ & 7.56 × 10^1^	5.85 × 10^1^ & 2.42 × 10^1^	120	5.5	90	36
Amoxicillin	1.6 × 10^2^	2.5 × 10^1^	10–20	13		35
Phenol	1 × 10^2^	1 × 10^2^	20	7	93.9	54

**Fig. 4:**
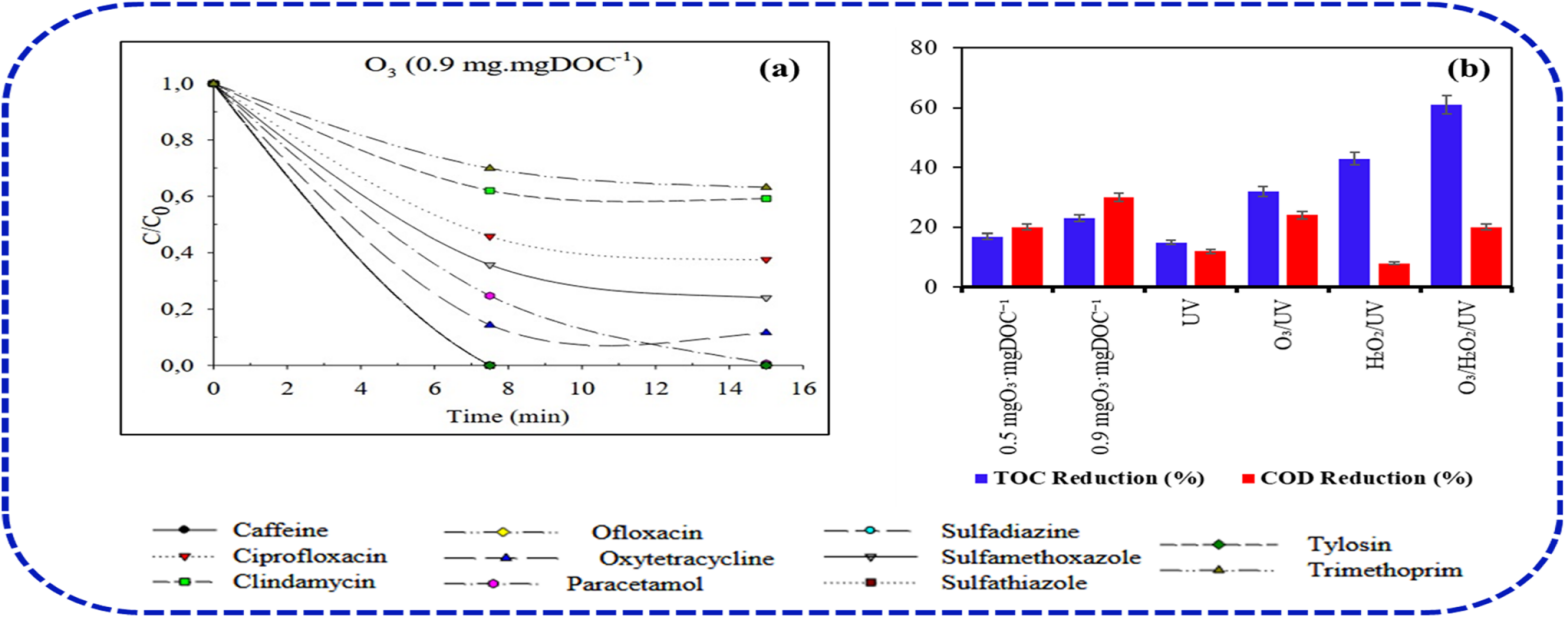
(a) Ozone dose effects on pharmaceutical degradation and (b) AOP comparison of COD/TOC removal from pharmaceutical effluent.^34^

In all cases, coupling ultrasonication with ozonation (US-1/O_3_ and US-2/O_3_) enhanced removal performance in which residual sCOD decreased to about 45–41%, and residual MBAS to approximately 17–13%, indicating synergistic effects between cavitation and ozone oxidation. The best sCOD and MBAS removal (residual ≈40%, and <10%) occurred for the highest ultrasonic energy and ozone dose, highlighting the interdependence of sonication time and O_3_ concentration. This improvement was attributed to ultrasonic shortening of surfactant chains followed by more effective mineralization during ozonation. Bisognin^34^ has demonstrated the performance of ozonation in the degradation of about 9 pharmaceutical wastewater contaminants at a neutral pH. The removal efficiency observed for oxytetracycline, caffeine, ciprofloxacin, and paracetamol at different ozone doses of 0.9 mg per mg DOC dissolved organic matter (DOC) after a contact time of 15 min shows about 89.32%, 96.79%, 97.75%, and 99.96%, respectively (Fig. 4a). The high degradation efficiency reflects both direct molecular ozone attack and enhanced radical formation with dissolved organic carbon, which further facilitates secondary reactions.^31^

**Fig. 5:**
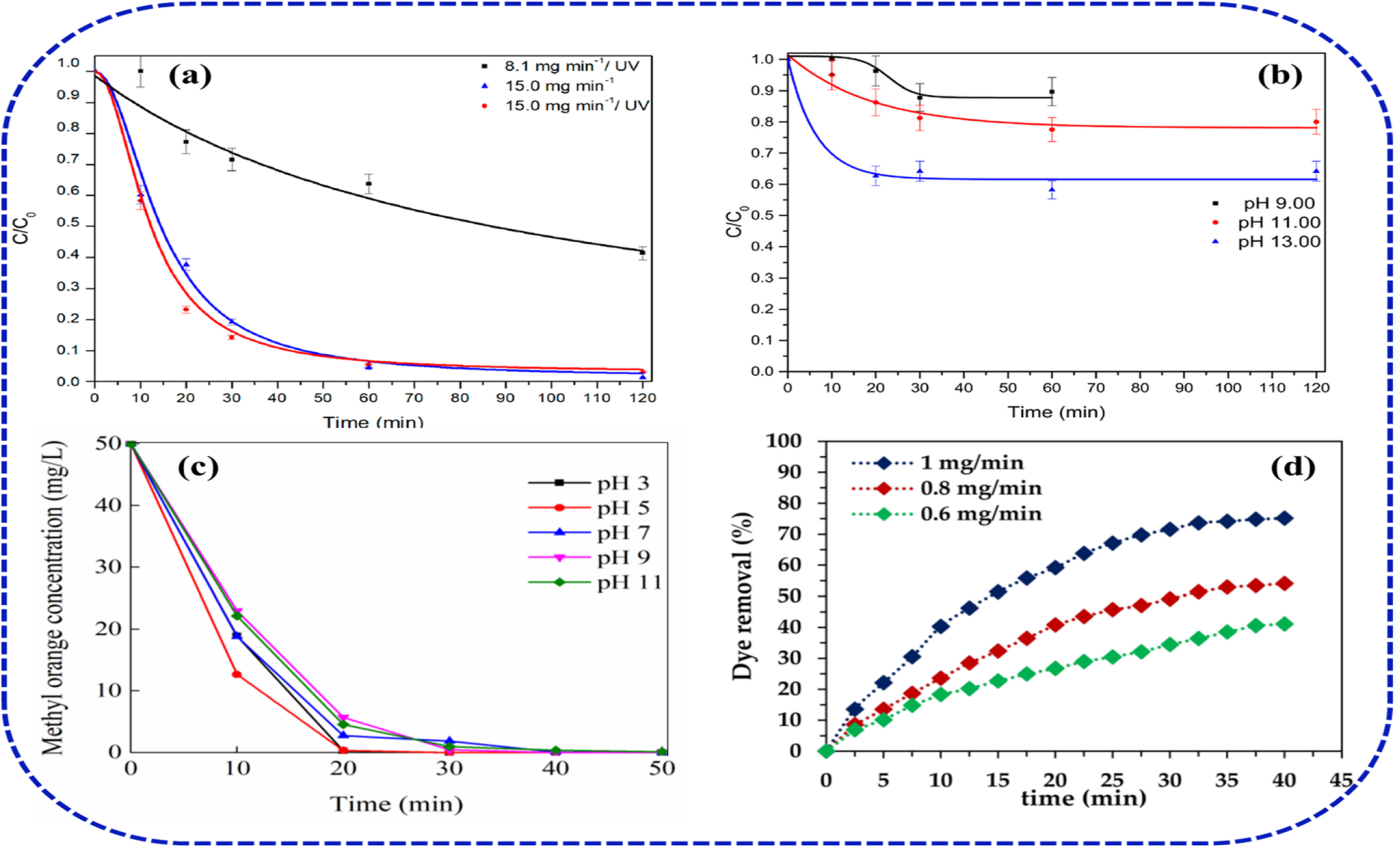
(a and b) Ozone dose, UV, pH effects on AMX removal, (c) MNB-enhanced MO ozonation, (d) Reactive Black 5 dose response.

Caffeine and paracetamol achieved near-complete removal, attributable to their relatively simple molecular structures that react readily with both ozone and HO˙ radicals. Similarly, they reported the COD and TOC mineralization of the pharmaceutical effluent using various configurations of AOPs with and without ozonation. The highest TOC reduction was recorded in the O_3_/H_2_O_2_/UV process, which presented average reduction efficiency of 60.52%, whereas the lowest performance was found in the UV process with 15.28% (Fig. 4b). Silva *et al.*^35^ have reported the effect of pH and capital analysis in the integration of UV in ozonation process for the degradation and mineralization of amoxicillin (AMX) from a pharmaceutical effluent. Based on their bench-scale kinetics, they argue that the gain provided by the integration of a UV unit in ozonation process does not compensate for the increase in capital and operational costs required for the addition of the UV irradiation equipment. In their experiment which adopted an ozone mass-rate of 8.13 mg min^−1^ and 15.00 mg min^−1^, both systems achieved almost the same AMX removal efficiency (Fig. 5a). They further noted that high pH ozonation gives the best cost-effective approach to degrade AMX (Fig. 5b). The kinetic investigation of AMX degradation by ozonation reported by Andreozzi, *et al.*^36^ shows that ozone attack is mainly directed towards the AMX phenolic ring leading to the formation of hydroxyderivative intermediates with no evidences on direct of attack on sulfur atom leading to sulfoxide formation. The effect of micro nanobubbles (MNBs) on the ozonation treatment efficiency of methyl orange (MO) was investigated by Xia and Hu.^37^ According to their experimental results, MO mineralization is greatly enhanced due to the combination with MNBs, in which the dye removal at a pH of 5 after 20–30 min was over 80–90%, respectively (Fig. 5c). In comparison to the similar investigation reported by Chen,^38^ the MO mineralization by ozone/MNBs process was found to be over two times higher than ozone, which demonstrated that MNBs can greatly enhance the treatment efficiency by ozonation.

The requirement for a high pH condition in the mineralization of organic contaminants has been expressed in mixed and somewhat contradictory arguments, where most reported studies indicate affirmative results. In the case of dyes and coloured contaminants, the effect of an increase in ozone dose is found to reflect the same behaviour in ozonation treatment efficiency.

**Fig. 6:**
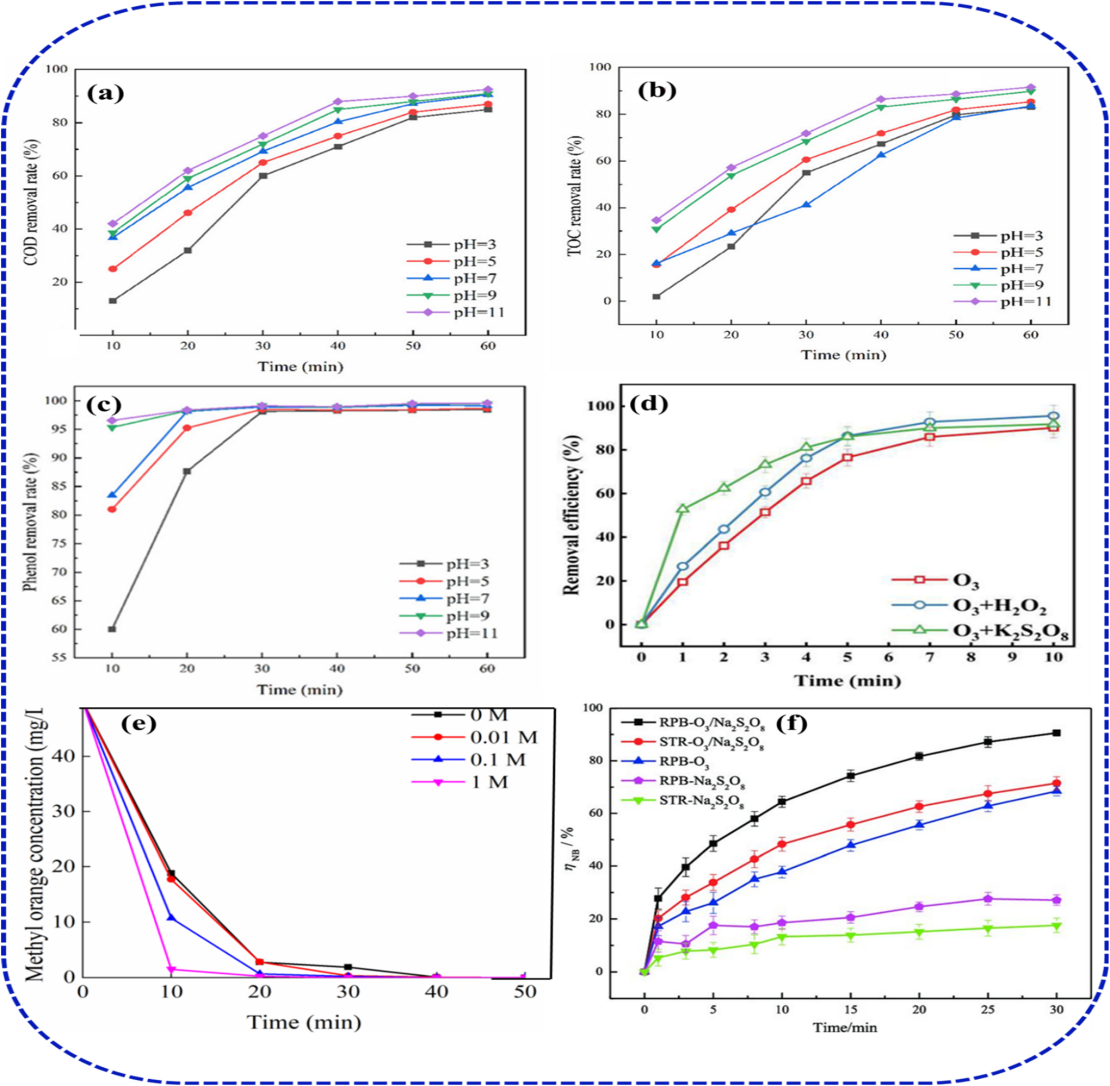
(a–c) pH effects on phenol/COD/TOC ozonation,^44^ (d) MO ozonation with persulfate^31^ (e) MO ozonation with salinity^37^ (f) ozonation of nitrobenzene (NB) with Na_2_S_2_O_8_.

Hussain^39^ has investigated the ozonation of Reactive Black 5 under different ozone concentrations and pH. They reported that effectiveness in dye removal increases with an increase in the ozone dose, in which the maximum efficiency (75.13%) was achieved at 1.0 mg per min ozone, compared to 0.8 mg min^−1^ (54.08%) and 0.6 mg min^−1^ (41.08%) doses, respectively (Fig. 5d). They suggested that at an alkaline pH, and a higher ozone dose, there is a rapid generation of hydroxyl radicals (˙OH) radicals, which subsequently enhances the rate of dye mineralization. Andreozzi *et al.*^36^ achieved about 90% amoxicillin and paracetamol degradation by ozone at a pH 5.5, ozone dose 1.6 × 10^−4^ M, and pollutant concentration 5.0 × 10^−4^ M, while Silva *et al.*^35^ documented high amoxicillin degradation (initial concentration 160 mg L^−1^) under higher alkaline and ozone rate conditions (pH 13, ozone rate 25 mg min^−1^). On the other hand, van Leeuwen *et al.*^50^ reported >90% degradation of methylene blue (MB) dye, at pH of 7 with an ozone rate of 0.5–1 mg L^−1^ after 10–20 minutes, confirming the effect of ionic nature and susceptibility of chromophoric groups of the contaminants to oxidative cleavage by ozone. Also, Tapalad *et al.*^40^ demonstrated that Congo Red (CR) dye ozonation achieved substantial colour removal up to 90% and COD reduction in a synthetic wastewater. The highest removal efficiency was observed in an alkaline condition, highlighting favourable radical generation and azo bond cleavage at higher pH.^41^ In comparison, Wulansarie^42^ reported even higher CR removal of 98% and significant reductions in COD at ozone flow rate of 400 mg L^−1^ after 60 minutes. Their results illustrate that increasing the solution pH intensely accelerates degradation and mineralization, due to the synergistic generation of hydroxyl radicals.^36^ Also, the effect of pH is particularly marked, with alkaline conditions favouring rapid depolymerization of dye molecules and facilitating conversion into low-toxicity products.^43^ The efficient degradation of phenol, COD, and TOC from simulated and real wastewater samples at a pH of 11 was reported by Zhou and co-workers.^44^ They demonstrated that the removal efficiencies for phenol, COD, and TOC in the simulated wastewater shows 99.60%, 96.58% and 90.33%, respectively (Fig. 6a–c). Meanwhile, the ozonation performance in the real sample at the same pH shows 80.63%, 69.75%, and 63.57%, respectively. They revealed that this performance is significantly better than that in acidic or neutral conditions. Their study further confirms the rapid solubility of ozone in alkaline medium to generate the reactive ˙OH (2.8 V), whose oxidizing capacity is more than that of molecular ozone (2.07 V), thus significantly enhancing the mineralization efficiency. The effect of coexisting ions and salts in the performance of ozone is also investigated to illustrate their enhancement and inhibitory activities. For instance, Xiang *et al.*^31^ demonstrated that performance of O_3_/H_2_O_2_ and O_3_/K_2_S_2_O_8_ systems, in which the maximum methyl orange (MO) removal rate of the two systems shows 95.6% and 91.8%, respectively (Fig. 6d). However, they reported that O_3_/K_2_S_2_O_8_ system performance within the first 5 min surpassed that of the O_3_/H_2_O_2_ system, which indicates that O_3_/K_2_S_2_O_8_ system exhibits stronger degradation performance in the short term. In the case of salinity effect on ozonation process, Xia and Hu^37^ have investigated the performance of ozone MNBs at various salinity conditions using sodium chloride (Fig. 6e). They indicated that 62% degradation was achieved after 10 min, in the 0 M salt system, whereas up on the addition of 0.1 M and 1 M of salt concentration into the system the MO degradation performance was enhanced to 78% and 96%, respectively. The addition of chloride was suggested to accelerate the dye oxidation at high salinity due to an increase in surface tension, resulting in higher internal pressure of the MNBs. Similar degradation results were reported by Wang^45^ and Yuan,^46^ who both indicated that, in the treatment of azo dye is enhanced at high chloride concentrations. This performance is also similar in the degradation of colourless organic contaminants. The effect of persulfate (Na_2_S_2_O_8_) in the ozonation treatment of nitrobenzene (NB) at a pH of 10 was reported to show 69.44% and 27.14%, in a standalone ozone and Na_2_S_2_O_8_ system, respectively (Fig. 6f). Whereas O_3_/Na_2_S_2_O_8_ integrated system achieved better efficiency at 90.59% after 30 min.^47^

Despite its powerful and versatile oxidation ability, ozonation faces significant technical, economic, and operational limitations that hinder its universal applicability. Its foremost constraint lies in the need for onsite ozone generation and inherent instability, demanding capital-intensive systems such as corona discharge or UV generators, air dryers, and off-gas destructors.^48^ High equipment maintenance and electricity consumption of about 15 kWh per kg O_3_ and strict safety requirements often make large-scale deployment of the ozonation process economically challenging.^49^ The ozonation process performance is highly sensitive to operational conditions, including temperature, pH, and the presence of organic matter radical scavengers, making it difficult to deal with wastewater containing varying water matrices and multiple co-existing contaminants.^20^ Additionally, wastewater treatment by ozonation can also generate toxic organic intermediates and unwanted by-products such as bromate under certain chemical conditions, posing regulatory and safety concerns. Thus, sustainable application demands careful kinetic modelling, real-time monitoring, and adaptive design to ensure regulatory compliance and long-term operational reliability.

## 3. Advances in photocatalysis process

Photocatalysis is a subset and one of the of advanced oxidation processes (AOPs) distinguished by the use of semiconductor materials (such as TiO_2_, CuO, ZnO, *etc.*) capable of harnessing light energy to initiate oxidation reactions.^55^ Upon UV or visible light excitation, the semiconducting photocatalyst absorbs photons with energy equal to or more than its band gap, generating electron–hole (e^−^/h^+^) pairs. These charge carriers migrate to the catalyst surface and drive redox reactions that generate ROS such as hydroxyl radicals (˙OH) and superoxide radicals (O_2_˙^−^).^56^ The highly reactive generated photocatalyst radicals further degrade organic contaminants adsorbed on the surface, often resulting in mineralization to CO_2_ and H_2_O.^57,58^ Enhancing the optical and photocatalytic properties of photocatalysts is critical for improving their efficiency and practical applicability under realistic solar irradiation.

### 3.1 Catalyst design strategies for enhanced photocatalysis

Recent advances in the process of photocatalysis and photocatalyst materials and systems are primarily aimed at overcoming the intrinsic limitations of conventional photocatalysts such as TiO_2_ and extending their practical applicability under solar irradiation and complex wastewater conditions. Several key strategies as summarised in Table 2 have been developed to achieve the specific goal of improving photocatalytic activities of semiconducting photocatalyst materials, including: doping with non-metal or metal elements.^58,59^ Formation of heterojunctions^60,61^ nano structuring and morphology control,^62^ sensitization by dye or plasmonic nanoparticles,^63^ and surface modification and functionalization.^64^ Collectively, these strategies significantly expand the photocatalysts' optical response toward the visible spectrum, enhance charge separation efficiency, and increase overall photodegradation performance.^65–67^ For example, doping with non-metals such as (N, S) or metals (Mn, Ag, Fe, Pd, Au) extends visible-light absorption by narrowing band gaps. Djaja^68^ reported the enhanced performance of manganese-doped ZnO and platinum-fluoride-modified TiO_2_ in the photocatalytic degradation of Malachite green dye. The doped samples demonstrate enhanced deactivation resistance and photodegradation rates of 75% and 65%, respectively. However, they also noted that excessive doping creates recombination centres that reduce quantum yields. While ensuring uniform dopant distribution reduces quantum yield, preventing leaching remains challenging for industrial scale-up.^69^ Heterojunction formation integrates semiconductors with complementary band structures, such as Fe-doped TiO_2_/SnO_2_ S-scheme composites or g-C_3_N_4_/TiO_2_, to facilitate charge separation and extend carrier lifetimes.^70^ These synergistic systems outperform individual components in degrading dyes and pharmaceuticals. Nevertheless, synthesis complexity, lattice incompatibility, and long-term durability under harsh wastewater conditions pose significant concerns.^71^ Nanostructuring through nanorods, nanosheets, and hierarchical porous architectures maximizes surface area and active sites. For example, a three-dimensional-based photocatalyst composite tends to exhibit superior mass transfer and charge mobility for photocatalysis air and water purification than its non-three-dimensional composite.^72^

**Table 2:** Modification strategies for enhanced photocatalytic performance of semiconductors

Strategy	Description	Rationale/key benefit	References
(1) Doping	Introduction of non-metals (N, S) or metals (Ag, Fe, Cu) into the crystal lattice	Band gap narrowing; improved visible-light absorption and charge carrier separation	81
(2) Heterojunction formation	Integration of two semiconductors with well-aligned band edges	Accelerated charge separation, prolonged carrier lifetime, and strong redox ability	82
(3) Nanostructuring	Design in forms of nanorods, nanosheets, or mesoporous/3D hierarchical architectures	Increased active surface area and enhanced light harvesting and mass transfer	83
(4) Plasmonic/dye sensitization	Attachment of plasmonic nanoparticles (Au, Ag) or organic dyes to the catalyst surface	Boosted visible-light response *via* plasmon resonance or photosensitization; more charge injection	63
(5) Surface modification/functionalization	Addition of functional groups (*e.g.* –COOH, –OH *etc.*), oxygen vacancies, or conductive coatings (*e.g.* PANI)	More reactive adsorption sites; improved electron mobility and ROS generation	84

Charge transfer hierarchy, particularly heterojunction systems, demonstrates superior photodegradation performance through preserved redox potentials and built-in fields, respectively. For instance, in a type-II heterojunction engineering, the composite operates through staggered band alignment, where photogenerated electrons migrate to the lower conduction band and holes to the higher valence band of adjacent semiconductors.^73^ This promotes spatial charge separation but weakens redox potentials; for instance, TiO_2_/CdS systems exhibit improved separation yet limited oxidative strength. This ensures effective spatial separation; however, it sacrifices redox potential, limiting the generation of highly reactive species.^74^ In contrast, a Z-scheme heterojunction mimics natural photosynthesis, where electrons from the conduction band of one semiconductor recombine with holes from another, preserving highly reductive electrons and strongly oxidative holes. A typical example is g-C_3_N_4_/WO_3_, widely reported for efficient pollutant degradation due to retained redox ability.^75,76^ Its key advantage lies in strong redox ability, though interfacial recombination losses and complex design remain limitations. Similarly, p–n junctions generate an internal electric field at the interface of p-type and n-type semiconductors (*e.g.*, CuO/TiO_2_), which drives directional charge migration and enhances separation efficiency. The emerging S-scheme heterojunction refines the Z-scheme concept by incorporating band bending and internal electric fields, allowing selective recombination of low-energy carriers while retaining high-energy electrons and holes; TiO_2_/g-C_3_N_4_ systems are frequently cited examples.^77,78^ While S-schemes offer an optimal balance between separation and redox strength, their mechanisms and long-term stability require further validation. Overall, Z-scheme and p–n systems are particularly advantageous in photocatalytic ozonation due to their ability to sustain strong redox potentials, thereby enhancing electron scavenging by O_3_ and maximizing reactive oxygen species generation over conventional oxidation pathways. Despite these advantages, precise synthesis techniques are costly and difficult to scale, while nanoparticle aggregation and potential ecological risks from nanoparticle release require careful management.^79^ Plasmonic and dye sensitization enhance visible-light responsiveness through noble metal nanoparticles (Au, Ag) that generate surface plasmon resonance effects, or organic photosensitizers targeting specific wavelengths. For instance, Au single-atom-anchored WO_3_/TiO_2_ nanotubes achieve remarkable VOC degradation efficiency compared to bare WO_3_/TiO_2_.^80^ However, photobleaching degrades dye stability, and noble metal costs combined with leaching concerns limit the scalability of the plasmonic and dye sensitization enhancement strategy.^75^ In surface modification enhancement strategy, oxygen vacancies, functional groups (such as –COOH, –OH), or conductive polymers are introduced to boost reactive oxygen species generation and electron mobility.^64^ While achievable through straightforward methods, surface instability over repeated cycles and recovery difficulties persist in this method. Thus, a successful enhancement photocatalyst design requires balancing enhanced activity with synthesis scalability, stability, and environmental safety, advancing toward sustainable, visible light, or solar-driven photocatalytic applications.

**Fig. 7:**
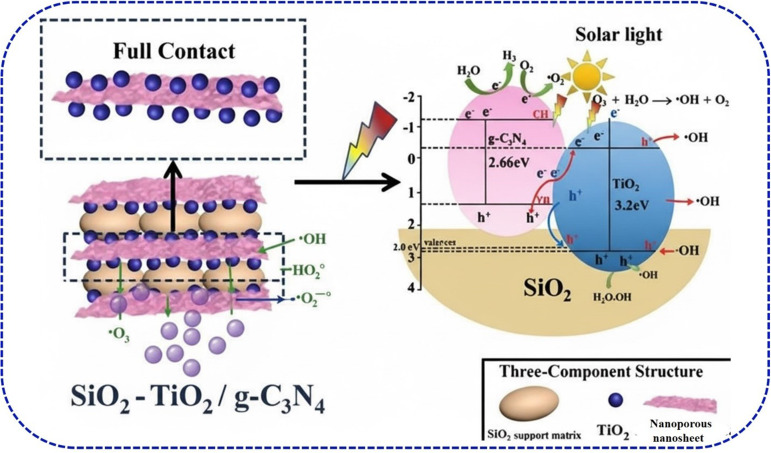
Enhancement of TiO_2_ degradation performance of RhB dye by heterojunction formation, adapted with permission from [Elsevier] ref. 87, copyright 2020.

Table 3 demonstrates how modified photocatalysts achieve effective organic dye degradation through optimized experimental conditions and strategic material design. Catalyst dosage, dye concentration, and light source critically determine performance. While increased catalyst loading enhances active site availability, excessive amounts cause light scattering and aggregation. Similarly, high dye concentrations impede light penetration and active site accessibility. The prevalence of sunlight and visible-light sources reflects practical applicability. Still, inherent band gap limitations necessitate material modifications for enhanced visible-light response. The photocatalysts employ synergistic approaches, doping, heterojunction formation, nanostructuring, plasmonic/dye sensitization, and surface modification, to address three fundamental challenges: expanding visible light absorption, suppressing electron–hole recombination, and increasing reactive sites. Despite improved efficiency, the data reveal practical challenges including extended irradiation times, high catalyst dosages, and increased material complexity and cost. These underscore the need for enhanced photocatalyst reusability, robustness optimization, and reactor design improvements for commercial viability.^85,86^ The construction of the ternary photocatalyst structure was investigated to prove the enhancement of longer carrier transport and resulting in a significant inhibition of photogenerated e^−^ and h^+^ recombination.^87^ prepared SiO_2_–TiO_2_/g-C_3_N_4_ composite photocatalyst using sol–gel and the removal rate of RhB by composites reached 98% after 180 min of solar-like irradiation, indicating that SiO_2_, TiO_2_, and CN had synergistic effect on performance improvement. The degradation mechanism illustrated in Fig. 7 demonstrated the interfacial existence of SiO_2_ and CN supporting the TiO_2_ formed in an efficient Z-scheme heterostructure. It can be observed that the amorphous SiO_2_ had a lot of active functional groups and a high surface activity, hence more TiO_2_ surfaces contact and combined with CN, could improve the dye degradation.

**Table 3:** Photocatalysis degradation of organic dyes using various modified photocatalysts

Catalysts	Organic dye	Conc. (mg L^−1^)	Light	Dose (mg L^−1^)	Time (min)	Efficiency (%)	Ref.
TiO_2_/CoCr_2_O_4_/SrTiO_3_	Basic yellow 28	12	Visible	2 × 10^2^	120	97	82
Fe_3_O_4_–TiO_2_–CoMoO_4_	Cresol red	20	Sunlight	4 × 10^2^	15–24	99.9	97
WO_3_–ZnO–NiO	Methylene blue	5	Sunlight	4 × 10^2^	105	45.87	98
Fe_3_O_4_–TiO_2_–CuO	Methylene blue	10	Visible	1 × 10^2^	25	99	99
ZnO–CuO–Ag_2_O	Rhodamine-B	20	Sunlight	2 × 10^2^	105	97.38	100
NiO–Fe_2_O_3_–CdO	Methylene blue	50	Visible	2 × 10^2^	60	84.25	101
MgO–Al_2_O_3_–ZnO	Methyl violet 6b	5	Visible	5 × 10^2^	210	93.42	102
ZnO–Fe_2_O_3_–MnO_2_	Methylene blue	30	Visible	1.5 × 10^2^	120	93	103
Mn_3_O_4_–ZnO–Eu_2_O_3_	Methyl orange	50	Sunlight	1 × 10^3^	150	96	104
Fe_2_O_3_–Mn_2_O_3_–FeMn_2_O_4_	Reactive Blue 222	20	Sunlight	5 × 10^3^	110	82	105
ZnO–MnO_2_–Gd_2_O_3_	Methylene blue	20	Visible	2.5 × 10^2^	150	94.4	106
Gd–BiFeO_3_	Rhodamine-B	20	Sunlight	3 × 10^2^	180	96	107
Zr–Ag_2_O	Rose bengal	10	Visible	2 × 10^3^	150	93.58	108
Ni-V_2_O_5_	Rhodamine-B	10	Visible	1 × 10^2^	140	99	109
Ni-WO_3_	Methyl red	10	Visible	2 × 10^2^	140	96	110

**Fig. 8:**
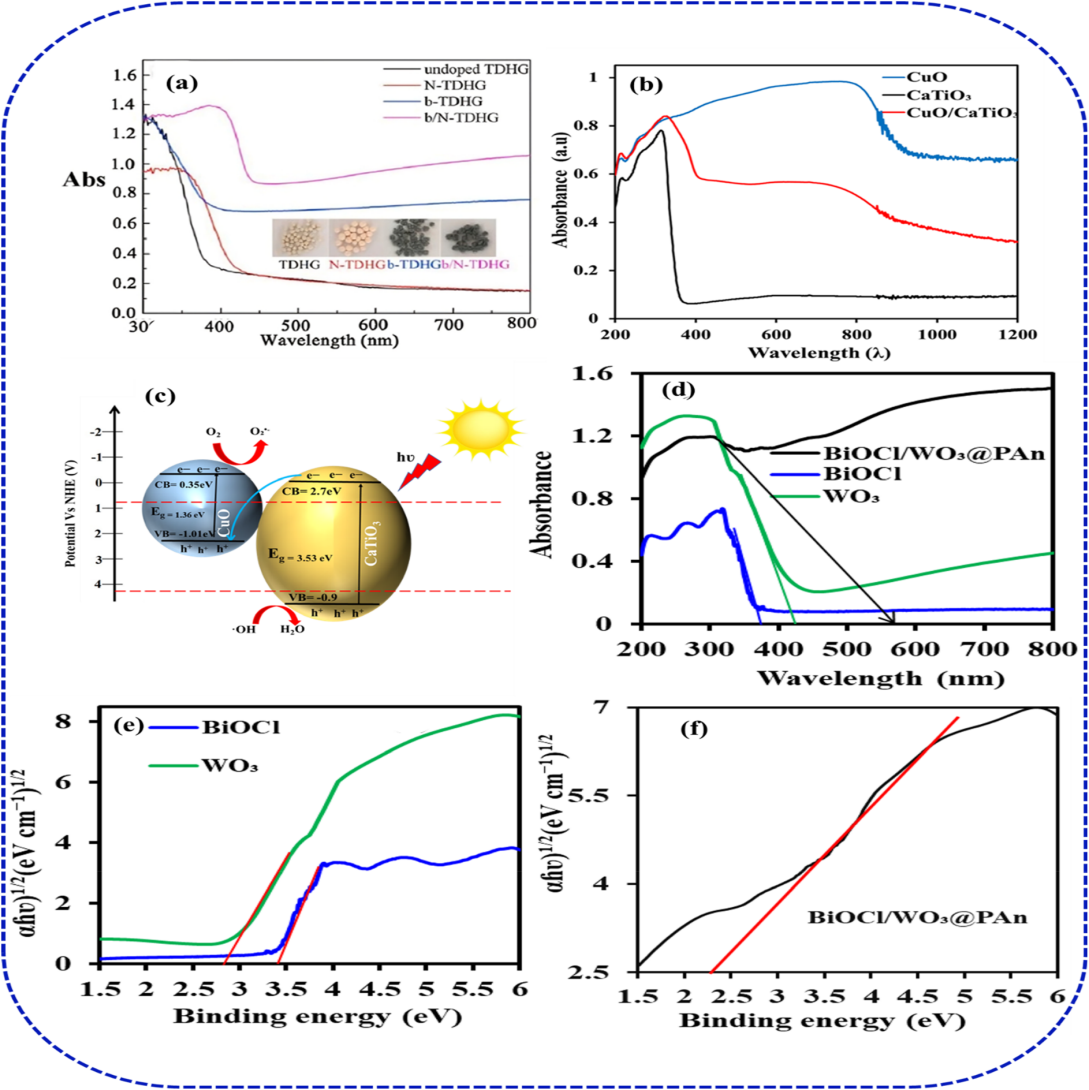
Visible-light absorption enhancement: (a) black Ti^3+^/N co-doped TiO_2_/diatomite, adapted with permission from [Elsevier] ref. 88, copyright 2014, (b and c) CuO/CaTiO_3_ heterojunction, adapted with permission from [Elsevier] ref. 93, copyright 2026, (d–f) BiOCl/WO_3_/PANI ternary.^76^

Similarly, Petala *et al.*^88^ reported the sol–gel synthesis of a black Ti^3+^/N co-doped TiO_2_/diatomite hybrid granule (b/N-TDHG) which exhibited a more pronounced light absorption than TDHG counterparts (Fig. 8a). The co-doped photocatalyst indicated more pronounced redshift and the strongest visible-light response resulted from the synergistic action of co-doped N and Ti^3+^. In our recent study, we constructed a strong built-in high optical absorption in CaTiO_3_ perovskite (band gap > 3.5 eV) with CuO heterojunction for enhanced solar-driven photocatalytic degradation of 2-chlorophenol in water.^89^ The new heterojunction CuO/CaTiO_3_ composite exhibited reduced photo absorption ability and reduced band gap (2.71 eV) with enhanced 2-CP degradation (99.28%), which is four times higher than the pristine CaTiO_3_ photocatalyst (Fig. 8b and c). As a p–n form of a heterojunction will be created as a result of joining the p-type CuO and n-type CaTiO_3_, the result demonstrates that visible light photo energy may excite both integrated semiconductors to produce photogenerated electrons and holes. Based on the above composite, there could be an exchange of electrons and holes between the two semiconductors as a result of the large difference in the Fermi energy levels.^90^ Conjugated polymers such as polyaniline (polymerised from aniline)^91^ have also received growing attention in recent years as a promising alternative to enhance the optical property and degradation performance of semiconducting photocatalyst. This is basically due to their low cost, high porosity, and high chemical stability.^83^ Kumar and co-workers^79^ demonstrated the facile fabrication of tungsten oxide (WO_3_)/bismuth oxychloride (BiOCl) immobilized on polyaniline (PAn) (BiOCl/WO_3_@PAn) heterojunction nanocomposite photocatalyst for the visible-light photodegradation of 2-CP. The highest 2-CP degradation (99.7%) was observed with the ternary composite due to enhanced photoadsorption ability brought about by the synergistic combination of PAn with WO_3_ and BiOCl (Fig. 8d–f). They suggested that the continuous increase in light absorption is mainly due to the polyaniline, which helps in the absorption of more photons from the solar spectrum, separating the produced e^−^ and h^+^, and generating more active radical species. In other scenarios, the addition of two or more non-metals or metal ions and/or a metal ion paired with a non-metal, literally referred to as co-doping,^92^ has been used to avoid imbalances of charges during the aliovalent doping process. Ultimately, these findings confirm that multidimensional photocatalyst engineering, integrating complementary modification strategies, effectively overcomes individual limitations, advancing solar-driven water treatment toward scalable, sustainable pollution remediation.

The basic process challenge in the photocatalysis degradation is the issue of limited optical utilization and the quick annihilation or recombination of photogenerated electron–hole pairs, which diminishes the effects of photocatalytic activity. Thus, semiconducting photocatalysts must be able to overcome these shortfalls provided that they meet the following conditions: (1) adequate or proper spectral absorption range, which is one of the main requirements of a given photocatalyst (2) appropriate band energy structure for the adequate separation and transport of e^−^–h^+^ pairs, and (3) satisfactory active sites to derive the adsorption or photocatalytic reaction.^94–96^ It is essential to satisfy all three above-mentioned prerequisites to improve photocatalytic efficiency, and abundant efforts have been made to systematically design photocatalysts and optimize photocatalytic dynamics.

## 4. Photocatalytic-ozonation (PC/O_3_) hybrid systems

Photocatalytic-ozonation (PC/O_3_) hybrid systems represent a more advanced approach that synergistically integrates the oxidative strengths of both photocatalysis and ozonation, providing an efficient platform for the degradation of persistent organic contaminants. This hybridization exploits the direct oxidation capability of ozone and the potent radical generation induced by semiconductor photocatalysts under light irradiation, primarily producing hydroxyl radicals that substantially accelerate pollutant mineralization beyond what either individual photocatalysis or the ozonation process alone.^24,111^ The enhanced generation of ROS results from ozone interacting with photogenerated charge carriers in the photocatalyst, thus increasing oxidizing efficiency and resulting in more radical yield.^10^ Moreover, photocatalysis aids in reducing ozone losses through decomposition by allowing sustained radical formation, thus maximizing ozone utilization for a longer duration.^49^ These synergistic mechanisms enable the treatment of diverse recalcitrant contaminants, including pharmaceuticals, dyes, and pesticides, often present in wastewater.

Experimental studies have also demonstrated that factors such as pH, catalyst type and loading, light intensity and wavelength, ozone dosage, and the hybrid reactor system design critically modulate the performance of PC/O_3_ hybrid system.^112,113^ Therefore, the optimization of these variables not only improves degradation kinetics but also enhances process stability and economic feasibility. For example, doped or heterojunction semiconductors facilitate charge separation, minimizing recombination, thus subsequently improving PC/O_3_ hybrid system performance compared to undoped or individual semiconducting photocatalyst.^84^ Furthermore, the hybrid process addresses limitations inherent to standalone ozonation or photocatalysis as earlier highlighted, such as incomplete mineralization, by-product formation, and energy inefficiencies.^42,114^ Hence, the PC/O_3_ hybrid system offers a promising route toward scalable, sustainable water treatment applications, with ongoing research focused on reactor engineering, catalyst development, and system integration to overcome operational challenges and reduce costs.

### 4.1 PC/O_3_ mechanisms and performance

**Fig. 9:**
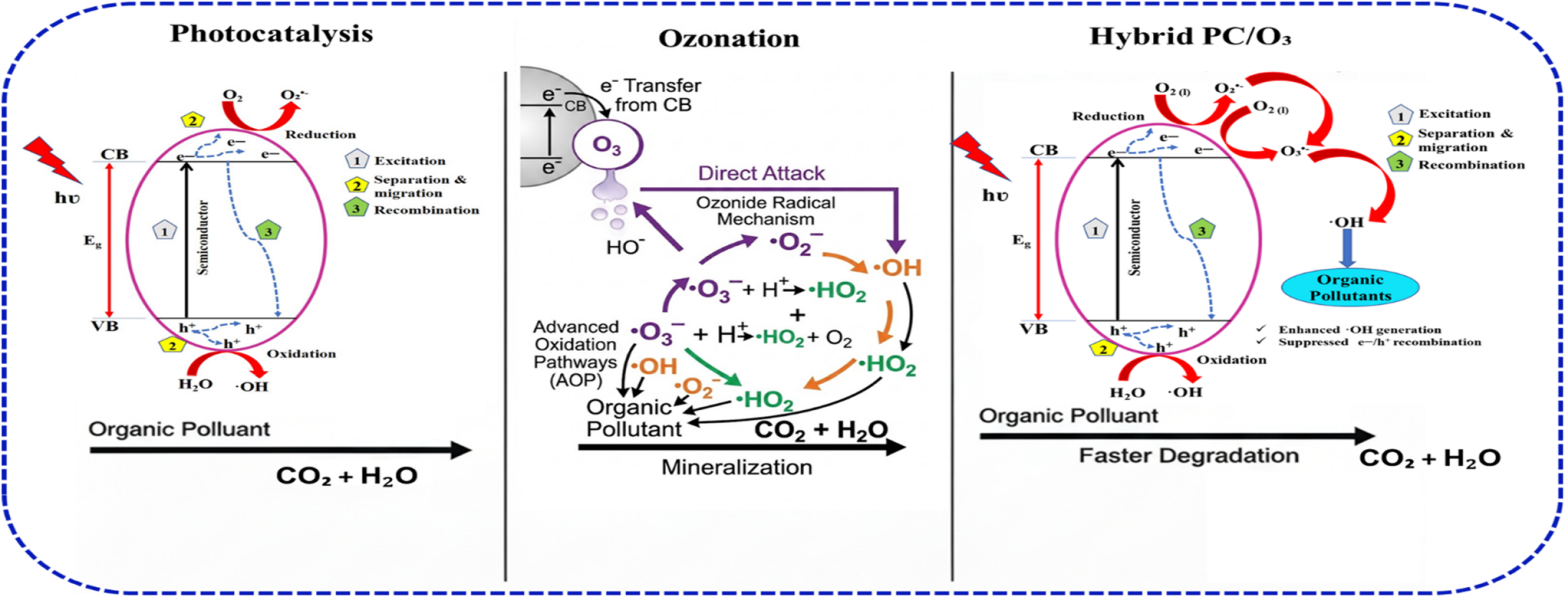
Mechanism of photocatalysis, ozonation, and hybrid photocatalytic-ozonation (PC/O_3_) in the generation of ROS.

Mechanistically, the PC/O_3_ process is governed by the concurrent or sequential generation of powerful oxidizing species, predominantly hydroxyl radicals (˙OH), *via* both photogenerated charge carriers (e^−^/h^+^) at the catalyst surface and ozone's decomposition pathways.^115–117^ In this co-existence of ozone with photocatalytic irradiation, multiple reactive pathways arise where ozone may directly oxidize organics, react with conduction band electrons to form superoxide anions (˙O_2_), or undergo catalytic decomposition to ˙OH on the surface, especially under visible or UV irradiation.^43^ Hence, in defining the oxidation mechanism attributed to PC/O_3_ (Fig. 9), it is important to note that the ozone serves as a highly efficient electron scavenger, significantly reducing the recombination rate of photogenerated electron–hole pairs and thus boosting the quantum efficiency of the photocatalyst.^24^ The improved charge carrier separation leads to elevated generation of ˙OH and ˙O_2_, resulting in faster and more extensive pollutant degradation compared to individual ozonation or photocatalysis alone. Furthermore, this PC/O_3_ hybrid approach facilitates the breakdown of ozone-resistant intermediates, which are often challenging in conventional ozonation.^118,119^ Performance-wise, PC/O_3_ systems have shown enhanced mineralization, and accelerated degradation kinetics, and reduced oxidant demand across a broad range of water matrices and contaminant classes. Photocatalytic holes (h^+^) oxidize water to form (˙OH) while electrons (e^−^) reduce O_3_ to O_3_˙^−^ and subsequently (˙OH) creating a complementary radical pathways. Ozone's high electron affinity (1.03 eV) promotes selective e^−^ scavenging, while surface h^+^ generate non-selective ˙OH for degrading recalcitrant pollutants.

**Fig. 10:**
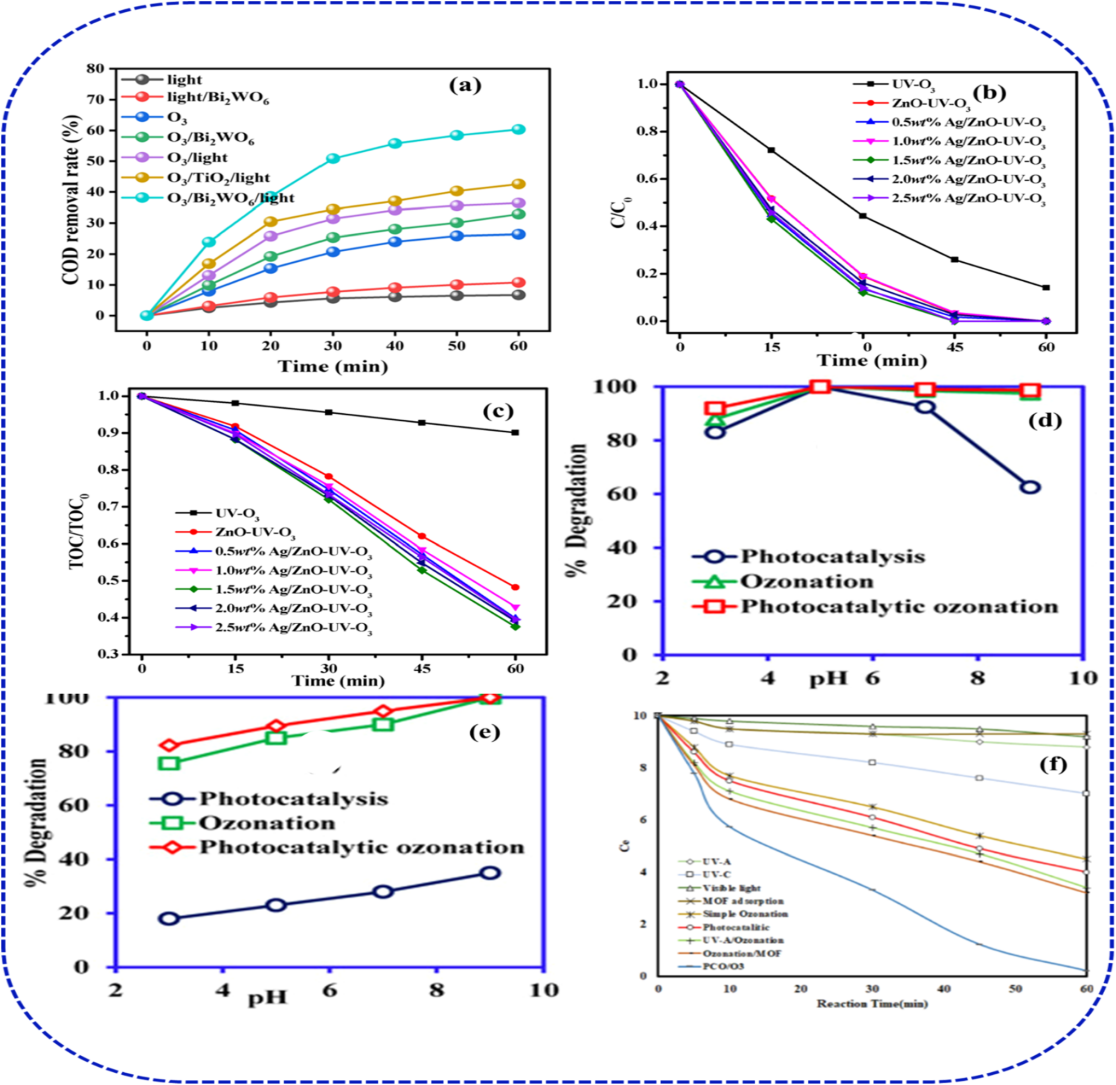
(a) PC/O_3_ performance of Bi_2_WO_6_ photocatalytic ozonation, adapted with permission from [Elsevier] ref. 119, copyright 2024, (b and c) COD removal from phenol-ammonia wastewater using ZnO & Ag/ZnO PC/O_3_ (ref. 120) (d and e) Ni–NiO/C/g-C_3_N_4_ dyes,^122^ (f) BiOI/NH_2_-MIL125(Ti) OTC, adapted with permission from [Springer] ref. 124, copyright 2023.

Different photocatalytic ozonation (PC/O_3_) studies have revealed a clear pattern of enhanced treatment efficiency driven by the synergistic interaction between photocatalysts, ozone dosage, and specific pollutant characteristics. For instance, Li *et al.*^119^ have demonstrated an enhanced COD removal from a coal chemical phenol-ammonia wastewater under various AOPs process configurations using two-dimensional nanosheets of Bi_2_WO_6_ as photocatalysts material (Fig. 10a). They revealed that the hybrid system containing Bi_2_WO_6_ photocatalytic ozonation exhibited highest performance due to a stronger O_3_ interaction force with W, which is easily activated by the surface-transferred electrons to form ROS and mineralize the organic pollutants. Their investigation concluded that ˙OH, ^1^O_2_, and ˙O_2_^−^ were responsible in the catalytic mechanism confirming the abundant generation of ROS during the photocatalytic ozonation oxidation compared to the single ozonation and photocatalysis processes. Similarly, Peng *et al.*,^120^ have studied the photocatalytic ozonation performance of ZnO and Ag/ZnO in different oxidation processes for the removal of phenol and TOD as demonstrated in Fig. 10b and c. They reported that Ag/Zn–UV–O_3_ process exhibited better performance nearly 100% for both phenol and COD removal with an average dose of 1.5 wt% of Ag, possibly due to a higher photoinduced carrier separation by the formation of Ag/ZnO heterostructure. They also noticed that the kinetics constant becomes slightly lower when more than 1.5 wt% of Ag is deposited with ZnO nanoparticles indicating the effect of over dosage of Ag catalyst in decomposing the ozone and impairing the ozonation process.^121^ Their investigation on the amount of Ag ion leaching shows about 0.004, 0.006, 0.009, and 0.004 mg L^−1^ in the four cycles, respectively, with no obvious change observed in the SEM image and the EDX analysis for Ag/ZnO after the reaction. Alhato *et al.*^122^ employed a Ni–NiO/C/g-C_3_N_4_ heterojunction (0.05 g catalyst) under visible light with an ozone flow rate of 20 g h^−1^, achieving rapid photocatalytic-ozonation of Congo Red and Alizarin Red S, as shown in Fig. 10d and e. Their experimental results show about 100% CR degradation efficiencies using the hybrid PC/O_3_ system at a pH of 5. Meanwhile, the degradation efficiency decreased in the alkaline condition due to the unfavourable surface interaction between the catalyst and the CR molecules, as an anionic.^123^ Also, the highest ARS degradation performance was achieved using PC/O_3_ system at an alkaline medium due to the cationic nature of the ARS dye unlike the acidic condition which can lead to an electrostatic repulsion between the dye and the catalyst. Mehralipour^124^ synthesized BiOI/NH_2_-MIL125(Ti) *via* solvo-thermal to investigate the photocatalytic-ozonation of oxytetracycline (OTC). Under optimum condition of pH 8.0, catalyst dose, 0.34 mg L^−1^, and O_3_ concentration of 28.7 mN, they reported about 96.2%, 77.2% and 64.2% removal of OTC, COD and respectively (Fig. 10f).

**Fig. 11:**
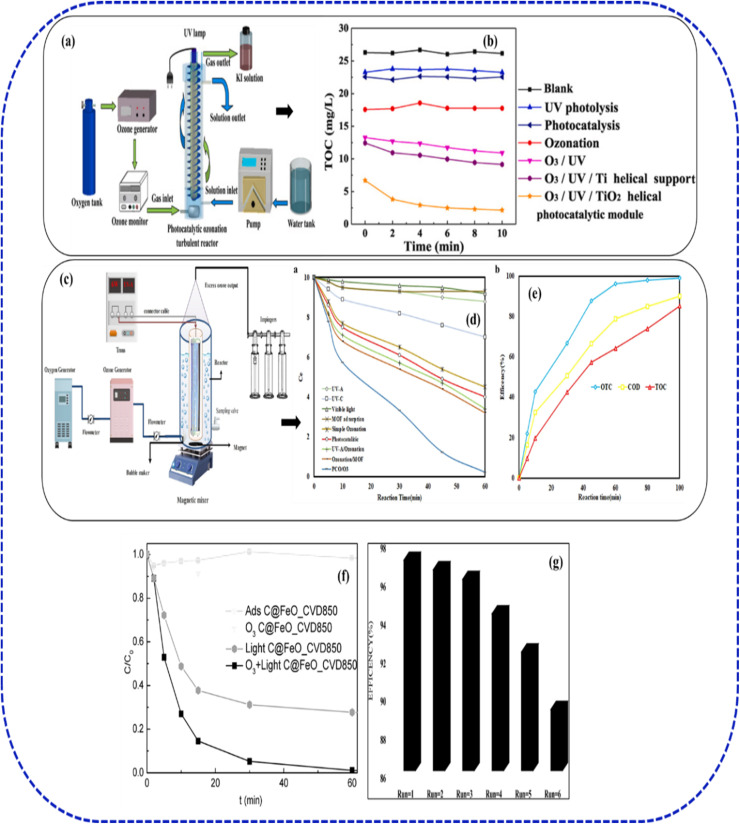
(a) Helical photocatalytic module (HPM) in an annular UVC reactor [Elsevier] ref. 127, copyright 2021, (b) UV-light assisted TOC reduction with TiO_2_ using HPM reactor, adapted with permission from [Elsevier] ref. 127, copyright 2021, (c) O_3_-HPCP catalytic reactor, adapted with permission from [Springer] ref. 124, copyright 2023, (d) degradation efficiency of PC/O_3_ using BiOI-MOF, adapted with permission from [Springer] ref. 124, copyright 2023, (e) OTC removal efficiencies using various AOPs systems, adapted with permission from [Springer] ref. 124, copyright 2023, (f) degradation efficiency of oxalic acid (OMA) using C@FeOCVD850,^128^ (g) stability of BiOI-MOF photocatalyst in removal of OTC, adapted with permission from [Springer] ref. 124, copyright 2023.

Unlike in the case of dye ionic properties, generally, as the increase in solution pH is more favourable for ozone decomposition rate and generation of free radicals, thus resulting in a higher removal efficiency. This observation is also reported by Asgari *et al.*,^125^ who also revealed the optimum photocatalytic ozonation process for ceftazidime removal using a pH value of 11. Unlike the case of dye ionic properties, increasing the solution pH generally favours ozone decomposition and the formation of reactive free radicals, thereby enhancing removal efficiency.^126^ Under alkaline conditions, ozone undergoes faster chain decomposition, generating hydroxyl radicals and other reactive oxygen species that promote non-selective oxidation of organic contaminants. This behaviour is consistent with the photocatalytic ozonation of Bi_2_WO_6_ systems, where the combined action of light irradiation and ozone improves charge separation, accelerates radical generation, and increases mineralization efficiency. Accordingly, Li *et al.*^119^ reported strong synergistic degradation performance using two-dimensional Bi_2_WO_6_ nanosheets composite in photocatalytic ozonation of organic pollutants in a coal chemical phenol–ammonia wastewater, supporting the view that alkaline conditions can be advantageous for ozone-assisted advanced oxidation processes. Kang *et al.*^127^ reported a benchmark of 95% UV-light assisted TOC reduction with TiO_2_ photocatalyst dosing at 20 mg L^−1^, ozone at 1 mg L^−1^ within 60 minutes in a neutral condition. They demonstrated enhanced PC/O_3_ mass-transfer using a helical photocatalytic module (HPM) within an annular UVC reactor (Fig. 11a). At a 19 min hydraulic retention time (HRT) and 26 mg L^−1^ influent TOC, HPM-integrated PC/O_3_ achieved 91.5% removal, vastly outperforming 58.1% *via* UVC/O_3_ (Fig. 11b). Their study illustrates the high efficiency of combining UV-activated TiO_2_ photocatalysts with a low ozone doses in a specialized reactor for relatively simple aromatic compounds. Furthermore, the hybrid process also provided an estimated energy-efficient PCO/O_3_ process with an output of 10.23 kWh per (m^3^ order), which is less than the magnitude per cubic meter of solution compared with other processes (24.30–68.75 kWh per (m^3^ order).^31^ To demonstrate the synergistic effect of the PC/O_3_ hybrid process,^128^ utilised a magnetic nanoparticles composed of iron oxide (FeO) cores) coated with carbon synthesised using chemical vapour decomposition (CVD) for the adsorption, photocatalysis, ozonation and PC/O_3_ of oxalic acid (OMA) which we selected as a model pollutant (Fig. 11c). They revealed that the photocatalyst (C@FeOCVD850) is not suitable for both adsorption and ozonation process removing only about 10–15% of OMA after 60 min of reaction. On the other hand, the OMA removal efficiency was improved under photoexcitation (about 70%) and demonstrated more significant performance using PC/O_3_ process (almost 100%), suggesting the presence of a synergy when O_3_, light and C@FeOCVD850 were acting simultaneously. Moreover, the verified PC/O_3_ efficiency during is higher than the sum of the performances obtained during photocatalysis and catalytic ozonation. Similarly, Mehralipour^124^ demonstrated the application of metal organic framework as photocatalysts using a solvo-thermally synthesised BiOI-MOF in the degradation of OTC through PC/O_3_ process using HPCP catalytic reactor. They achieved about 96.2% OTC removal and 77.2% total COD reduction a catalyst does of 0.34 mg L^−1^ with an ozone concentration of 28.7 mg L^−1^ under optimized conditions of pH 8.0 and 56 minute reaction time (Fig. 11d–f). They also noted that ozone concentration required for PCO/O_3_ hybrid process solely depends on the type of reaction reactor, the type of pollutant, and the specifications of the intermediate compounds. Regarding the OTC and COD degradation at a pH of 8, they further confirmed that oxygen radicals are favourably generated when a photocatalyst is combined with ozone under alkaline conditions. Thus, when ˙O_2_^−^ radicals are present in water, they create ˙OH radicals, which eventually enhance ozonation efficiency.^49^ Also, their investigation on the effect of ozone gas concentration (20–40 mM L^−1^ min^−1^) shows that increasing the ozone gas concentration also increases efficiency of the process followed by a slight decrease at a higher ozone concentration. With the increase dissolved ozone concentration, mass transfer in the reaction media also increases leading to a synergistic effect, reactive oxygen species (ROS), particularly hydroxyl radicals, are produced in greater quantities. The BiOI-MOF photocatalysts exhibited strong stability achieving degradation performance of about 89.2% after sixth consecutive cycles (Fig. 11g), suggesting the efficient performance and economic advantage of the MOF photocatalyst in the PC/O_3_ hybrid process. It is obvious that most potential photocatalysts employed for PC/O_3_ hybrid process demonstrates a significant stability and excellent performance after several degradation cycles, implying an insignificant interference of the ozone with the catalyst chemical structure.

An intermittent or continuous dosage of the ozone gas at times affects the performance of the PC/O_3_ process, depending on the reactor and the photocatalyst employed. The synergistic effect of photocatalysis and ozonation in the mineralization of 10 mg L^−1^ of MB and MO dyes was also investigated from the study reported by Nabizadeh *et al.*^129^ They demonstrated that the combined effects of lighting and ozonation have proved enhanced decolorization and degradation of the pollutants using TiO_2_ and ZnO nanoparticles with ozone supplied either intermittently or continuously by a SDBD cold plasma reactor. The most significant decolouration results were obtained using an intermittent ozonation process, which achieves 97% degradation of MB based on TiO_2_ catalysis. On the other hand, ZnO nanoparticles achieved only 40% degradation of MO, demonstrating the effect of the difference in ionic dyes in the catalytic process. Their study also presents the efficiency of even pristine large band gap intrinsic photocatalyst such as TiO_2_ in achieving improved degradation under the hybrid PC/O_3_ process. Asgari *et al.*^130^ have also demonstrated the photocatalytic ozonation system consisting of O_3_/UVA/TiO_2_ in the removal of 10 mg L^−1^ concentration of ciprofloxacin antibiotic (CIP) from its aqueous solutions. The catalyst achieved 98% in the first run, and the efficiency was about 95% after 6 recycling steps. Similarly, thin film photocatalyst have been employed in the hybrid PC/O_3_ system. Avramescu *et al.*^131^ investigated paracetamol removal *via* photocatalytic ozonation over TiO_2_–CeO_2_ and TiO_2_–SnO_2_ thin films under UV radiation, achieving improved mineralization compared to individual processes. Overall, these studies affirm that photocatalytic ozonation efficiency is modulated by catalyst type and dose, ozone levels, pollutant chemistry, and experimental pH and contact time. Enhanced charge carrier dynamics through doping or heterojunctions and the amplified hydroxyl radical generation through O_3_ photolysis support the superior performance over standalone processes. Another ciprofloxacin antibiotic (CIP) removal with concentration of 10 mg L^−1^ was also reported by Marathe^132^ using 5 wt% V_2_O_5_/ZnO photocatalyst achieving about 94% at neutral pH and catalyst loading of 1 g L^−1^. The systems performance in 15 min of contact time at various pH levels was about 86.4%, 92.2%, 94%, 86.8%, and 87.2% for pH of 3, 5, 7, 9, and 11, respectively. The COD removal was highest at pH 7, reaching 80% in 30 min with no further removal at the end of 120 min. Similarly, their energy consumption analysis at optimised conditions suggested that the PC/O_3_ hybrid process fared well compared to individual AOPs. The effectiveness of the PC/O_3_ hybrid system in the degradation of mixed pharmaceutical pollutants was also demonstrated by Jamil^133^ who reported about 86.9% COD removal from pharmaceutical wastewater containing carbamazepine and amoxicillin using modified clay/TiO_2_/ZnO at 1 g per L catalyst and 0.6 mg per min ozone dose. However, depending on the photocatalysts and other experimental conditions, some PC/O_3_ require longer reaction to achieve better results. For example, Liao *et al.*^134^ employed BiPO_4_ nanorods for sodium dodecyl benzene sulfonate degradation under UV/O_3_ conditions, achieving 90.0% TOC removal at 75 minutes, although demonstrating superior performance compared to standalone conventional TiO_2_ photocatalysis. The results demonstrated that the TOC removal rate of the UV/O_3_/BiPO_4_ process was dramatically 4.9 and 3.8 times more than that of UV/BiPO_4_ and O_3_. Overall, these studies provide evidence that photocatalytic ozonation represents a promising technique for water treatment as indicated from various presented in Table 4. The high efficiencies across pharmaceutical and dye pollutants underline PC/O_3_'s promise in real-world water treatment, provided operational parameters are finely controlled. For example, lower ozone dosages with highly active catalysts balance energy and material costs,^135^ while longer contact times and appropriate pH adjustment tend to favour complete mineralization of persistent organic compounds.^31^

**Table 4:** Degradation of various organic wastewater using hybrid photocatalytic-ozonation process

Photocatalyst	Photocatalyst dose (mg L^−1^)	O_3_ rate (mg L^−1^)	Light	Pollutant	Conc. (mg L^−1^)	pH	Time (min)	Eff. (%)	Reference
TiO_2_	2 × 10^4^	1	UV	Phenol	50	7	60	∼95	127
N-doped TiO_2_	2 × 10^4^	0.5	UV	MB	30	6.8	45	∼90	59
TiO_2_/CuO	1 × 10^4^	0.8	Visible	Malachite	10	7	30	75	68
g-C_3_N_4_/TiO_2_	4× 10^4^	1	Visible	Phenol	20	7	40	90	60
Au–TiO_2_	3 × 10^4^	0.7	UV	VOCs	5	7	120	95	80
ZnO–Pd	1 × 10^4^	1.2	Visible	Ciprofloxacin	10	7	25	85	41
Fe-doped TiO_2_/SnO_2_	5 × 10^0^	0.9	UV	Reactive	15	6.5	35	80–85	60
Mn-doped ZnO	3 × 10^4^	1.1	Visible	Phenol	50	7	50	75	68
Pt-fluoride TiO_2_	35	1	Visible	Malachite	8	7.5	20	65	68
Ag–ZnO/GO	2 × 10^4^	0.6	Visible	MB	30	7	45	90	63
Fe_3_O_4_–TiO_2_–CoMoO_4_	4 × 10^4^	0.7	Sunlight	Cresol	20	7	15	100	59
Ni-V_2_O_5_	1 × 10^4^	0.7	Visible	Rhodamine	10	6.5	140	99	63
MgO–Al_2_O_3_–ZnO	5 × 10^4^	0.6	UV	MB	5	7	210	93	68
BiOI/NH_2_-MIL125(Ti)	2.87 × 10^1^	0.34	—	Oxytetracycline		9	56	96.2	124
TiO_2_/SnO_2_	6 × 10^1^	1	UV	Sulfamethoxazole	10	7	30	85	21
V_2_O_5_/ZnO	1 × 10^4^	—	Visible	Ciprofloxacin		7	—	—	132
Clay/TiO_2_/ZnO	1 × 10^4^	0.6	—	Carbamazepine & amoxicillin		7	—	86.9	133
TiO_2_–CeO_2_ and TiO_2_–SnO_2_ thin films	—	—	Visible	Paracetamol		—	—	—	131

**Table 5:** Regeneration and photocatalytic performance of some photocatalyst materials

Catalyst	Pollutant	Cycles	Activity retention	Leaching data	Regeneration method	Ref.
TiO_2_	Phenol	5	92% (5th cycle)	Not reported	Washing	179
Ag–ZnO	Phenol	6	95% (6th cycle)	Not reported	Ultrasonic cleaning	120
ZnO/TiO_2_	Phenol	7	90% (5th cycle)	Not reported	Thermal treatment	180
Mn-ZnO	Malachite green	5	82% (5th cycle)	2.1 mg L^−1^	Acid leaching	68
BiOI-MOF	OTC	4	88% (4th cycle)	<0.1 mg L^−1^	Ethanol washing	124

### 4.2 Factors affecting PC/O_3_ process

**Fig. 12:**
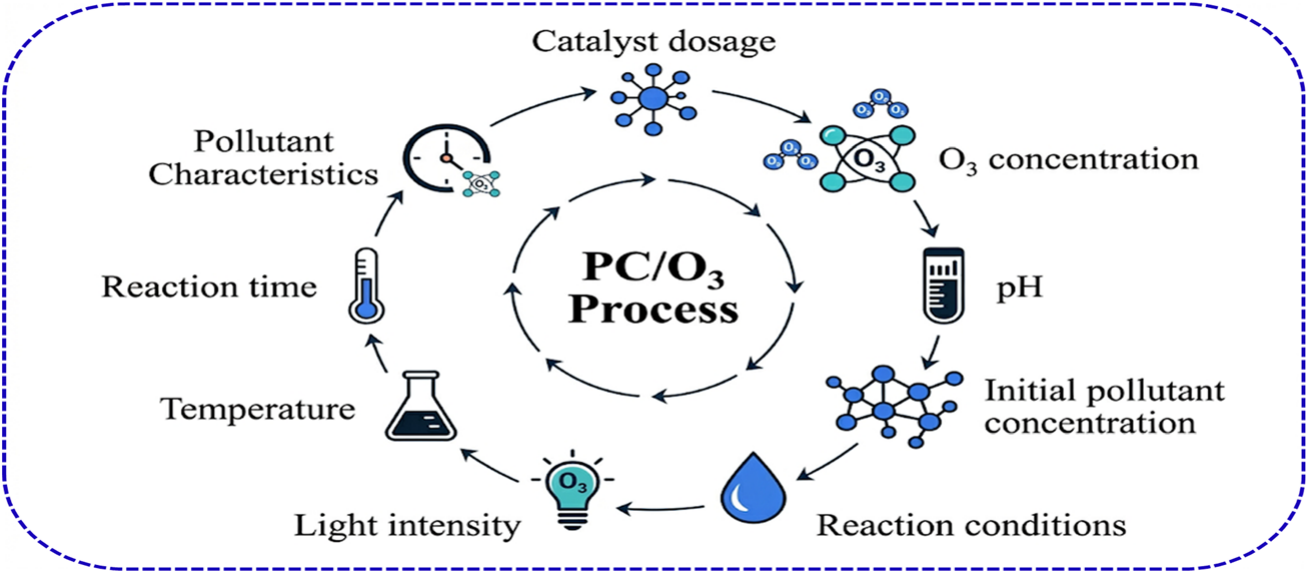
Key factors influencing the photocatalytic ozonation (PC/O_3_) process.

The performance of the hybrid photocatalytic-ozonation process typically depends on several interconnected variables including operational parameters such as catalyst features, ozone concentration influencing reactive species, pollutant properties, and reaction kinetics *etc.* (Fig. 12). Operating parameters in PC/O_3_ play a crucial role in governing hydroxyl radical production and the overall oxidation efficiency. It represents the most readily adjustable variables in PC/O_3_ systems and exert profound influence on both photocatalytic degradation kinetics and ozonation efficiency.^134,136^ The optimization of these parameters requires balancing often-competing objectives: maximizing pollutant degradation while maintaining high ozone oxidation ability and lifetime to ensure complete mineralization of the target pollutants.

#### 4.2.1 Catalyst dosage

Catalyst dosage in PC/O_3_ processes exhibits a dual effect that is critical to optimizing high degradation performance. For instance, increasing the amount of photocatalyst enhances the availability of active sites for both ozone activation and light absorption, thereby promoting the generation of ROS.^15,137^ This effect enhances interaction between catalysts, ozone molecules, and light energy, thus increasing the efficiency of pollutant mineralization.^138^ However, above an optimum catalyst loading threshold, excess particles tend to agglomerate, leading to reduced effective surface availability and increased light scattering.^17^ This scattering limits photon penetration into the reactor, hence could diminish the catalyst's photoactivation efficiency.^139^ Furthermore, higher catalyst concentrations can enhance electron–hole recombination rates, which reduces the number of charge carriers available to initiate ozone reduction and radical formation.^140–142^ This cumulative effect ultimately lowers the overall photocatalytic activity. Therefore, it is necessary to optimize the catalyst dosage, typically ranging from tens to hundreds of milligrams per litre depending on reactor configuration and operational parameters. Reported results of statistical design of experiments often reveal that an optimal catalyst dosage balances these competing phenomena, maximizing pollutant degradation and mineralization rates.^129^

#### 4.2.2 Ozone concentration

The concentration of ozone is one of the most dominant factors in PC/O_3_ advanced oxidation treatment system. It is obvious that increasing ozone dosage initially boosts ROS generation by reacting with photogenerated electrons to form ozonide radicals,^143^ which subsequently generate hydroxyl radicals.^144^ However, excessive ozone can scavenge radicals or shield light penetration, adversely affecting catalyst activation. In most case, the photocatalyst easily adsorbs dissolved ozone molecules because of weak hydrogen bonds with their surface ˙OH groups. Anions of ozonide radicals are produced by capturing electrons on the surface of the photocatalyst. Thus, the ozonide radicals (O_3_˙^−^) increase the amount of ˙OH radicals and the efficacy of pollutant degradation.^124^ However, when the incoming ozonation flow increases excessively, and the mass transfer rate from the gas phase to the liquid phase is limited, the efficacy of degradation also decreases. Photocatalytic ozonation studies earlier reported have reported similar findings, which agree with this concept. Thus, an optimum ozone concentration must be established, often determined experimentally for each system.

#### 4.2.3 Solution pH

The solution pH markedly influences PCO/O_3_ mechanisms in which acidic to neutral pH favours ozone stability but limits radical formation,^145^ while alkaline conditions promote the decomposition of ozone, yielding more hydroxyl radicals for enhanced degradation.^146^ However, extreme alkaline pH conditions could also induce radical scavenging by bicarbonates and carbonates, weakening pollutant degradation performance.^147^ It is well established that the pH also alters pollutant and catalyst surface charges, modifying adsorption and electron transfer.^148^

#### 4.2.4 Light intensity and wavelength

The intensity of the photo irradiation significantly affects PCO/O_3_ treatment system as photocatalyst activity is directly proportional to the level of photoactivation.^149^ Thus, sufficient photon flux matching the catalyst band gap is essential for electron–hole pair generation, which results in ozone reduction and radical formation.^150^ Different research studies have reported the enhancement of visible or UV-activated catalysts in the development of solar-driven PCO/O_3_.

#### 4.2.5 Catalytic reaction time

The length of catalytic reaction time in photocatalytic ozonation systems is obviously reduced compared to individual photocatalysis and ozonation processes due to synergistic effects between photogenerated charge carriers and ozone-derived reactive oxygen species. The hybrid system accelerates pollutant degradation by providing multiple oxidation pathways simultaneously, with PC/O_3_ achieving more than 90% removal of recalcitrant organic pollutants within 15–45 minutes, compared to 90–180 minutes required for conventional photocatalysis or ozonation alone.^151^ The enhanced efficiency stems from rapid ozone activation by photogenerated electrons, producing superoxide and hydroxyl radicals, while photogenerated holes directly oxidize pollutants.^152^ Reaction time optimization typically ranges from 10–60 minutes, depending on pollutant complexity, catalyst properties, and ozone dosage. These shorter durations reduce operational costs and energy consumption, making PCO/O_3_ economically viable for industrial wastewater treatment applications requiring rapid turnover rates.^84^ The studies reported earlier have demonstrated that catalytic reaction time critically influences PC/O_3_ process efficiency, where the optimal treatment durations vary by depending on employed experimental conditions. For example, Mehralipour *et al.*^153^ found that extending reaction time from 30 to 60 minutes significantly enhanced tocilizumab removal, achieving 92% degradation and about 79.8% total COD reduction under optimal conditions. However, beyond optimum intervals, diminishing returns emerge as pollutant concentrations increase. Similarly, oxytetracycline degradation studies revealed that while adequate reaction time improves removal, extensions beyond catalyst or ozone radical depletion offer less significant advantage. Bukhari *et al.*,^154^ observed progressive improvements in total COD and nitrogen removal with increasing photocatalytic-ozonation reaction time in simulated hospital wastewater. Mehling *et al.*^155^ confirmed influence of PC/O_3_ superior kinetics, reducing the reaction time for micro-pollutant degradation by nearly three-fold compared to standalone processes, which achieved same efficiency after 80 minutes for photocatalysis and ozonation processes alone. These findings establish that while extended reaction time generally promotes contaminant degradation and mineralization in the hybrid PC/O_3_ systems, process-specific optimization determined by pollutant characteristics, catalyst properties, and oxidant availability play significant roles in gaining enhanced removal outputs.

#### 4.2.6 Photocatalyst characteristics

Catalyst properties critically govern PCO/O_3_ performance by influencing light absorption, charge separation, and ozone activation. High surface area nanostructured catalysts with optimized morphology enhance pollutant–catalyst interactions, tend to provide abundant active sites, and improve mass transport.^113,156^ Enhancement of photocatalyst strategies, such as band gap tuning, enables efficient visible light absorption and promotes electron transfer to ozone, generating reactive oxygen species.^157^ Also, the creation of surface defects in photocatalyst, oxygen vacancies, and strategic doping enhances charge carrier separation while creating preferential adsorption sites for both ozone and pollutants.^158^ The formation of composite heterojunctions and hybrid systems combining metal oxides with carbon materials or noble metals establishes synergistic charge transfer pathways, amplifying catalytic efficiency.^159,160^ Above all, the catalyst stability under simultaneous irradiation and ozone exposure is essential to prevent deactivation through structural degradation or surface fouling.^161^ Ultimately, strategic catalyst design tailored to specific pollutants and operational conditions is vital for achieving optimized, sustained PCO/O_3_ system performance.

#### 4.2.7 Pollutant characteristics

The chemical nature and physical properties of pollutants critically dictate PC/O_3_ hybrid system reactivity and degradation efficiency. Pollutants molecular structure of electron-rich aromatic compounds, such as phenols, and amines undergo rapid mineralization through facile radical attack, while saturated hydrocarbons exhibit slower degradation kinetics.^95,162^ Additionally, pollutant concentration inversely affects removal efficiency, as elevated loads saturate catalyst active sites and reduce radical availability, with accumulating intermediates competing for oxidative species.^163^ Solubility and hydrophobicity influence catalyst surface adsorption such that hydrophobic pollutants may require surface-modified catalysts or surfactant enhancement.^164,165^ Therefore, understanding pollutant matrix complexities, including concentration ranges, functional groups, and interfering substances, enables tailored PCO/O_3_ system design for effective wastewater and industrial effluent treatment. While O_3_ electron scavenging and ˙OH enhancement represents dominant PCO_3_ synergies, literature also reports surface ozonide (O_3_^−^˙) and non-radical pathways (^1^O_3_). Pathway dominance depends on catalyst surface properties, ozone concentration, and pH, though comprehensive EPR/scavenger diagnostics remain limited. For an in-depth understanding of reaction pathways and underlying mechanisms, reference can be made to comprehensive mechanistic reviews available in the literature.^166–168^ Matrix effects systematically limit mineralization in which real wastewaters averagely exhibit about 45% lower TOC removal than synthetic solutions across AOPs due to radical scavenging (humics, bicarbonates), oxidant competition (multiple pollutants), and inorganic inhibition due to anions such as Cl^−^, SO_4_^2−^*etc.*^74^ In study reported by Zhou *et al.*,^44^ under optimal conditions, ozonation of simulated phenol wastewater (50 mg L^−1^) achieved a maximum COD removal of 90.60%. In contrast, for real phenol-spiked wastewater under identical conditions, removal efficiencies were lower, reaching 65.45% (phenol), 63.57% (COD), and 79.65% (TOC). This, study clearly demonstrates nanobubble mitigation of salinity scavenging. Hence, PCO_3_ system synergy significantly overcomes matrix limitations through dual oxidant pathways even though it requires a tighter parameter optimization.

## 5. Challenges and limitations of the PC/O_3_ systems

Despite its promising performance, the practical implementation of PC/O_3_ encounters still encounters several challenges. These challenges span from process integration mechanisms, catalyst issues, reactor design constraints, scalability, as well as energy, and environmental and regulatory concerns. Therefore, addressing these challenges and limitations is vital for advancing PC/O_3_ from laboratory research to scalable widespread industrial application.

### 5.1 Optimization and process integration challenges

The process of integrating PC/O_3_ hybrid system requires the consideration of multiple operational parameters that need careful balancing to maximize synergistic effects without counterproductive interactions. Most importantly, the simultaneous control of pH, catalyst loading, ozone dosage, reaction time, and light intensity presents a complex multi-variable optimization problem.^169^ For instance, catalyst or ozone overdosing may generate radical scavenging effects and subsequently reduce the easy penetration of photons due to an increased catalyst aggregation and turbidity.^21^ Contrary to a case of insufficient doses, which could lead to a reduced generation of reactive species.^170,171^ Furthermore, proper synchronization between photocatalyst activation and ozone mass transfer is critical, as inefficient photon delivery and ozone dissolution onto the catalyst surface may limit sufficient radical production.^135^ In the case of real wastewater samples, heterogeneous nature of pollutants containing inorganic ions and organic matter further complicates PC/O_3_ system optimization due to pollutant-specific radical scavenging.^172^ Hence, advanced statistical design and modelling of experiments with real-time monitoring tools are still evolving to accurately predict and control these variables for efficient PC/O_3_ performance at pilot and industrial scales.

### 5.2 Catalyst stability and regeneration

The chemical durability of most catalysts employed for PC/O_3_ processes remains a significant limitation for its successful application. This is because the exposure to strong oxidative radicals generated during PC/O_3_ oxidation process could induce photo-corrosion and structural degradation in chemically unstable semiconducting catalysts, reducing their activity over repeated cycles.^173^ Additionally, the problem of surface fouling due to organic intermediates or inorganic deposits, such as carbonates and phosphates, may also block available active sites.^174^ For metal or non-metal doped photocatalyst, dopants and co-catalysts intended for visible-light activation may leach or restructure to other complexes under combined UV/ozone treatment.^175^ Thus, an effective regeneration protocols comprising of promising techniques such as photo-assisted cleaning, thermal treatment, and chemical washing are required to restore catalytic activity, even though they add more complexity and operational cost.^176,177^ TiO_2_-based catalysts generally maintain >90% activity after 5–7 cycles *via* simple washing/calcination, while metal-doped systems show higher activity loss. Regeneration methods include ultrasonic cleaning (most common), ethanol washing, and thermal treatment, though surface area loss and active site poisoning mechanisms remain unquantified. The results from most investigated studies reveals that typical PCO_3_ catalysts maintain up to 95% activity after 4–7 cycles, with TiO_2_-based materials showing superior stability *versus* metal-doped systems. Leaching ranges from negligible (<0.1 mg L^−1^) for BiOI-MOF^124^ to 2.1 mg per L Mn from Mn-ZnO,^68^ raising environmental concerns. Regeneration strategies include ultrasonic/ethanol washing (most common, >95% recovery), acid leaching (effective but risks structural damage), and thermal calcination which is best for most carbon-based catalysts. Analytical research gaps still remain with only less than 20% of studies reported provided quantitative ICP-MS leaching, quantify surface area loss post-cycling, and bromate formation monitoring data. Also, comprehensive life-cycle assessment and regulatory leaching limits (*e.g.*, WHO drinking water standards) remain unaddressed in most PCO_3_ literature. Spent PCO_3_ photocatalyst regeneration aligns with the sustainable development goals (SDGs) 12 which aims to ensure sustainable consumption and production patterns through closed-loop recycling strategies. However, disposal challenges persist where heavy metal leaching particularly from Ag and Mn based composite photocatalysts exceeding WHO limits^178^ during regeneration risking to secondary contamination (SDG 6), along with nanoparticle release ecotoxicity concerns (SDG 14). Thus, sustainable pathways include the utilization of non-metal catalysts such as g-C_3_N_4_, use of waste-derived photocatalysts materials and magnetic recovery composites such as Fe_3_O_4_, eliminating filtration and supporting SDG 9 on innovation. Current literature lacks life-cycle assessments quantifying embodied energy, regeneration chemical use, and end-of-life disposal impacts, representing a critical gap for SDG-aligned commercialization.

### 5.3 Reactor design and scalability limitations

Besides the complexity in the design of PC/O_3_ reactor, scaling and process control from laboratory to industrial applications also pose notable challenges. The optimal PC/O_3_ hybrid system reactor configurations often feature thin catalyst layers and efficient light penetration,^111,172^ whereas ozonation requires effective gas–liquid mass transfer equipment to maximize ozone utilization.^178^ Integrating both photocatalysis and ozonation simultaneously without incurring significant hydrodynamic or mass transfer limitations demands innovative reactor designs. In particular, larger-scale systems may suffer from light attenuation and non-uniform catalyst irradiation, reducing overall quantum efficiency.^149,177^ To overcome this challenge, practical solutions, including ensuring adequate photon flux across larger volumes and immobilizing catalysts while maintaining high surface area and mechanical stability, are usually employed.^179,180^ Detailed scale-up models based on radiative transfer, chemical kinetics, and fluid dynamics remain under development to provide design guidelines. Although several reactor configurations have been reported for PCO_3_, the literature remains predominantly laboratory-scale, and clear pilot-scale design rules are still limited. The main scale-up bottlenecks include gas–liquid ozone transfer, light attenuation in larger volumes, catalyst separation, and non-uniform hydrodynamics.^181^ According this, larger photocatalytic reactors suffer from reduced photon penetration and that thin catalyst layers are preferred to maintain light utilization, while effective ozone delivery requires strong gas–liquid mass transfer.^182^ In this context, the helical photocatalytic module (HPM) used by Kang *et al.*^127^ is a useful example, since its annular configuration was designed to improve irradiation efficiency and support a measurable TOC reduction under PCO conditions. Similarly, Mehralipour *et al.*^124^ applied an HPCP catalytic reactor for OTC removal and reported 96.2% OTC removal and 77.2% COD reduction at pH 8, showing that reactor configuration can strongly affect performance when ozone transfer and catalyst contact are optimized. Flow-oriented designs are also supported by the reported use of continuous or intermittent ozone dosing. Nabizadeh *et al.*^129^ found that intermittent ozonation improved MB removal to 97% with TiO_2_, indicating that reactor operation and gas delivery mode can materially affect hybrid efficiency. For catalyst handling, immobilized or thin-film systems are attractive because they simplify recovery and reduce post-treatment separation, as shown by the TiO_2_–CeO and TiO–SnO thin films used for paracetamol degradation.^131^ Overall, the available studies suggest that practical reactor design should prioritize photon utilization, ozone dissolution, and catalyst accessibility rather than simply increasing reactor volume; however, comprehensive pilot-scale data, radiative-transfer models, and validated scale-up correlations remain scarce in the current PCO_3_ literature.

### 5.4 Economics and environmental regulatory concerns

The hybrid PC/O_3_ systems basically face economic challenges from its energy-intensive ozone generation and the integration of UV/visible light-only activated catalyst, alongside capital costs for specialized reactor design and equipment.^181^ While the synergistic effects enhance pollutants removal efficiency and reduce treatment times, on the other hand suboptimal operation increases energy waste. Life cycle assessments have indicated that an optimized PC/O_3_ hybrid system can be cost-competitive for treating recalcitrant pollutants, especially if solar-driven catalysts and energy-efficient ozone generation are utilized.^183^ Environmental compliance requires managing incomplete oxidation intermediates, ozone residues, and catalyst leaching to prevent secondary contamination. Furthermore, ozone's toxicity necessitates careful handling and off-gas destruction.^184^ The data reported in Table 6 reveals PCO_3_'s distinct techno-economic position among AOPs, balancing energy efficiency with hybrid system complexity. Kang *et al.*^127^ benchmarked TiO_2_ PCO_3_ at 10.23 kWh per m^3^ order for phenol degradation, around 50% lower than standalone photocatalysis (between 20–100 kWh per m^3^ order) and competitive with ozonation (equivalent ∼15 kWh per kg O_3_ at up to 90% gas transfer efficiency). This energy advantage stems from ozone's electron scavenging, reducing e^−^/h^+^ recombination while photocatalysis enhances O_3_ utilization, creating synergistic ROS production at lower total energy input. Similarly, Li *et al.*^185^ reported sulfamethazine degradation by UV/H_2_O_2_ with EE/O down to ∼0.06 kWh per m^3^ order, demonstrating high efficiency and energy optimization. Botelho Ruas *et al.*^186^ apply UV/H_2_O_2_–anaerobic hybrid treatment to complex wastewater organics, lowering effective kWh m^−3^*via* biological polishing. The study reported by Mehralipour *et al.*^124^ on PC/O_3_ targets oxytetracycline, optimizing energy consumption (EEC) while increasing mineralization *versus* stand-alone ozonation or photocatalysis. Generally, PCO_3_ capital expenditure splits across catalyst (25%), ozone generator (35%), and reactor (40%), requiring balanced upfront investment *versus* single-process simplicity. This energy-cost profile of PCO_3_ based on operating cost (OPEX) favourably positions it as a best choice for recalcitrant pollutants (pharmaceuticals, dyes) degradation where standalone processes fail, even though scale-up economics remain challenging. However, pilot studies demonstrate viability at 1–10 m^3^ h^−1^ outputs, yet full-scale validation data for >100 m^3^ h^−1^ are scarce.^187^ Thus, on the basis of life cycle assessment (LCA), considerations favour PCO_3_ over Fenton due to its no iron sludge advantage but highlight the risks of metal leaching and bromate formation potential under alkaline conditions. Future commercialization demands solar-driven PCO_3_ (eliminating the cost of UV) as major capital cost (CAPEX) driver, robust catalyst design (reducing replacement frequency), and integrated reactor optimization (maximizing ozone transfer).^190–193^

**Table 6:** Energy consumption and cost comparison across AOPs

Process	Energy metric	Reported range	Capital cost drivers	OPEX drivers	Example	Reference
Ozonation	kWh per kg O_3_	7–18 kWh kg^−1^	Ozone generator (40–60% CAPEX)	Electricity (50–70% OPEX)	N/A	188
Photocatalysis	kWh per m^3^ order	20–100 kWh m^−3^	UV lamps/reactor (60%)	Lamp replacement (40%)	N/A	189
PCO_3_	kWh per m^3^ order	10–25 kWh m^−3^	Catalyst + ozone + reactor (50%)	Ozone generation (60%)	10.23 kWh m^−3^ (phenol, TiO_2_)	120

## 6. Operating-condition trends across the reviewed studies

Table 7 shows that each of the three processes (photocatalysis, ozonation, PCO_3_) performs best within an optimum operating window rather than under unlimited increase in operating intensity. In ozonation particularly, higher ozone dose generally improves removal, as seen for Reactive Black 5, where increasing the ozone rate from 0.6 to 1.0 mg min^−1^ raised degradation from 41.08 to 75.13%, and for phenol, COD, and TOC, where alkaline pH 11 markedly improved removal in both simulated and real wastewater^39^ However, the gain is not always proportional, since Silva *et al.*^35^ reported that UV addition to ozonation did not significantly improve amoxicillin removal relative to ozone alone, despite higher capital and operating cost. In photocatalysis, the reviewed studies demonstrate that photocatalyst dose must also be optimized and modified photocatalysts outperform bare materials. On the other hand, excessive catalyst loading can limit light penetration and reduce efficiency by scattering and agglomeration. For hybrid PCO_3_, the best performance is obtained only when ozone dose, catalyst loading, irradiation conditions, and wastewater matrix are jointly balanced; this is supported by the reported high removals for hybrid systems. However, it has been observed that real wastewater experiments showed lower mineralization than model solutions because of matrix scavenging and competing organics. Also, averagely PCO_3_ consistently showed superior TOC/COD removal efficiency (70–95%) and lower energy demand (10–12 kWh per m^3^ order) over ozonation (60–97%, 15 kWh per kg O_3_) and photocatalysis (40–85%). Meanwhile, a direct pollutant comparison of all the reported studies indicates a non-linear optimum. For instances in phenolic degradation, Zhou *et al.*^44^ achieved 99.6% phenol, 96.6% COD, 90.3% TOC (simulated wastewater, pH 11) *vs.* 80.6% phenol, 69.8% COD, 63.6% TOC (real wastewater) using ozonation, showing strong matrix effects. Kang *et al.*^127^ reported 95% TOC with TiO_2_ PCO_3_ at 1 mg per L ozone, 20 mg per L catalyst, 60 min. Peng *et al.* achieved 100% COD with Ag–ZnO PCO_3_ compared to about 75% degradation for a standalone process. In the case of dye degradation, Hussain *et al.* showed Reactive Black 5 ozonation increasing from 41% (0.6 mg min^−1^) to 75% (1.0 mg min^−1^), demonstrating dose optimum, while Djaja *et al.*^68^ reported 75% Malachite green with Mn-ZnO photocatalysis, limited by light scattering at higher loadings. The positive effect of a high pH in ozonation process is indicated from the study reported by Silva *et al.*^35^ which found amoxicillin ozonation (pH 13, 25 mg min^−1^) achieving near-complete removal with minimal UV addition benefit, while Bisognin *et al.*^34^ reported >90% oxytetracycline and caffeine degradation at pH 7 using 0.9 mg per mg DOC.

**Table 7:** Operating parameter trends across ozonation, photocatalysis, and PCO_3_ processes

Method	Oxidant dose	Catalyst loading	Irradiation conditions	TOC/COD removal (mg L^−1^)	Energy metrics	Wastewater matrix characteristics	Overall operating balance
Ozonation	Higher ozone dose improves removal up to an optimum	Not applicable	Not applicable	TOC: 60–90%; COD: 70–97%	15 kWh per kg O_3_	Real wastewater lowers efficiency due to scavengers and competing organics	Performance depends on dose and matrix balance
Photocatalysis	Not applicable	Optimum loading is required; excess causes scattering	Light must match the catalyst band gap	TOC: 40–80%; COD: 50–85%	Not widely reported	Matrix species suppress active sites and radical utilization	Efficiency depends on balancing loading, light, and pollutant concentration
PCO_3_	Ozone dose must be optimized to avoid scavenging	Catalyst loading must be optimized to avoid shielding	Irradiation must sustain charge separation	TOC: 70–95%; COD: 75–90%	10–12 kWh per m^3^ order	Matrix effects are stronger in real effluents	Best performance needs simultaneous optimization of all operating factors

## 7. Conclusions and future perspectives

This review has demonstrated the effectiveness of ozonation, photocatalysis, and hybrid photocatalytic ozonation (PCO_3_) in the degradation of organic wastewater contaminants. The comparative analysis shows that no single standalone AOP is universally superior; rather, process selection should be driven by pollutant type, matrix complexity, treatment goal, and energy demand. Ozonation is most suitable when fast oxidation and simple operation are required, but its performance is strongly governed by pH and wastewater composition. Photocatalysis is attractive for solar-driven treatment, yet its practical use remains limited by light utilization, catalyst recovery, and the need for high-performance visible-light materials. PCO_3_ provides the strongest overall treatment performance when ozone dose, catalyst loading, irradiation conditions, and reactor design are jointly optimized, particularly for recalcitrant industrial effluents. Thus, a hybrid PC/O_3_ system emerges as a highly promising advanced oxidation process, synergistically harnessing the strengths of both photocatalysis and ozonation to achieve enhanced removal of recalcitrant organic pollutants. Catalyst advancements, including strategic doping, heterojunction formation, and tailored morphologies, substantially improve light utilization, charge carrier separation, and long-term stability, furthering the effectiveness of PC/O_3_ wastewater treatments. Experimental results consistently confirm the system's ability to degrade challenging contaminants such as pharmaceuticals, dyes, and pesticides, often attaining over 90% mineralization under favourable conditions. However, a widespread adoption of the PC/O_3_ faces several persistent barriers, including the demands of process integration and scaling, catalyst deactivation and regeneration, high energy consumption, and the complexities of reactor and system design. Additionally, environmental and regulatory considerations, such as catalyst sustainability and the mitigation of hazardous by-product formation, still remain significant.

Looking forward to addressing these critical issues demands research efforts. Key practical application research priorities should focus on continuous-flow reactor design, improved ozone mass transfer, catalyst immobilization or magnetic recovery, and validation in real wastewater rather than only synthetic solutions. More studies should report TOC/COD removal, electrical energy per order, catalyst lifetime, leaching, and regeneration efficiency so that performance can be compared on a common basis. In addition, pilot-scale studies are needed to determine whether laboratory-level synergy remains effective under realistic hydrodynamic and optical constraints. The development of rigorous kinetic and mechanistic models to support process optimization for real-world wastewaters is also required for the incorporation of renewable energy, notably solar irradiation and energy-efficient ozone generation, in proving the promise that would enhance the sustainability and economic feasibility of the PC/O_3_ system. Comprehensive risk assessments and life cycle determination, alongside advancements in system automation and real-time monitoring, are also crucial for reliably deploying PC/O_3_ technologies. In summary, advanced oxidation process of wastewater decontamination, particularly photocatalysis, ozonation, and most importantly, hybrid PC/O_3_ have the capacity to become a foundation of advanced wastewater remediation, if supported by continued research, responsible implementation, and collaborative development within the environmental technology community.

## Conflicts of interest

On behalf of all authors, the corresponding author states that there is no conflict of interest in this work.

## Data Availability

No primary research results, software or code have been included and no new data were generated or analysed as part of this review.
